# Applicability of Single-Layer Graphene as a Hydrogen-Blocking Interlayer in Low-Temperature PEMFCs

**DOI:** 10.1021/acsami.4c01254

**Published:** 2024-04-27

**Authors:** Miriam Komma, Anna T. S. Freiberg, Dunia Abbas, Funda Arslan, Maja Milosevic, Serhiy Cherevko, Simon Thiele, Thomas Böhm

**Affiliations:** † Forschungszentrum Jülich GmbH, Helmholtz Institute Erlangen-Nürnberg for Renewable Energy (IEK-11), Cauerstr.1, 91058 Erlangen, Germany; ‡ Department of Chemical and Biological Engineering, Friedrich-Alexander-Universität Erlangen-Nürnberg, Cauerstr.1, 91058 Erlangen, Germany

**Keywords:** single-layer graphene, fuel cell, low-temperature proton exchange membrane fuel cell, proton exchange membrane, hydrogen gas crossover, gas barrier layer, 2D materials

## Abstract

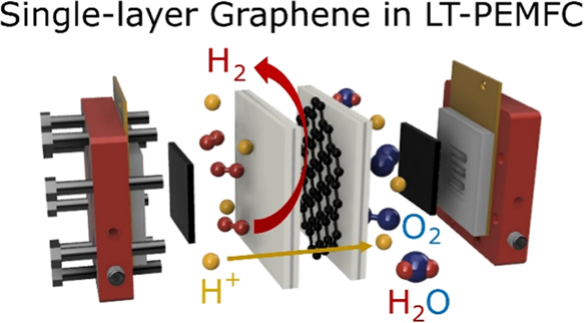

Gas crossover is critical in proton exchange membrane (PEM)-based electrochemical systems. Recently, single-layer graphene (SLG) has gained great research interest due to its outstanding properties as a barrier layer for small molecules like hydrogen. However, the applicability of SLG as a gas-blocking interlayer in PEMs has yet to be fully understood. In this work, two different approaches for transferring SLG from a copper or a polymeric substrate onto PEMs are compared regarding their application in low-temperature PEM fuel cells. The SLG is sandwiched between two Nafion XL membranes to form a stable composite membrane. The successful transfer is confirmed by Raman spectroscopy and in ex situ hydrogen permeation experiments in the dry state, where a reduction of 50% upon SLG incorporation is achieved. The SLG composite membranes are characterized by their performance and hydrogen-blocking ability in a fuel cell setup at typical operating conditions of 80 °C and with fully humidified gases. The performance of the fuel cell incorporating an SLG composite membrane is equal to that of the reference cell when avoiding the direct etching process from a copper substrate, as remnants from copper etching deteriorate the performance of the fuel cell. For both transfer processes, the hydrogen crossover reduction of SLG composite membranes is only 15–19% (1.5 bar_abs_) in the operating fuel cell. Further, hydrogen pumping experiments suggest that the barrier function of SLG impairs the water transport through the membrane, which may affect water management in electrochemical applications. In summary, this work shows the successful transfer of SLG into a PEM and confirms the effective hydrogen-blocking capability of the SLG interlayer. However, the hydrogen-blocking ability is significantly reduced when running the cell at the typical humidified operating conditions of PEM fuel cells, which follows from a combination of reversible interlayer alteration upon humidification and irreversible defect formation upon PEM fuel cell operation.

## Introduction

Fuel cells (FCs) and water electrolyzers can contribute to reducing carbon emissions in the future.^1,2^ Both their efficiency and lifetime need to be optimized for successful large-scale commercialization. Decreasing fuel crossover—and therewith increasing the faradaic efficiency—in electrochemical energy conversion devices is crucial for obtaining highly efficient systems and reducing safety concerns,^3−6^ particularly in water electrolyzers.^7,8^ In addition, minimizing the crossover of hydrogen, oxygen, or alcohol through the electrolyte in fuel cells increases the system’s lifetime, as degradation effects on membrane electrode assemblies (MEAs) are lessened^3−6,9^ by, e.g., reducing the formation of radicals in proton exchange membrane fuel cells (PEMFCs).^3,4^ Barrier layers of different materials have been investigated to reduce crossover.^5−7,10−12^ For PEM applications, suitable barrier layers need to maintain the proton conductivity of the membrane while blocking the crossover of fuel and product gases.

A damage-free SLG has been proposed as an ideal blocking layer, as it is known to be a close-to-perfect barrier layer for molecules and nearly all atoms at ambient conditions.^13−16^ Additionally, Hu et al.^16^ showed that SLG is capable of conducting protons, making it a promising material for barrier interlayers in PEMs. Furthermore, the authors also found that the proton conductivity of SLG directly correlates with temperature. The combination of the superior sieving properties of SLG and its proton conductivity led to several studies about the potential use of SLG as a crossover-reducing barrier layer in FC applications.^5,10,11,17−19^

Yan et al.^10^ successfully used SLG as a barrier layer for methanol in a passive direct methanol fuel cell (DMFC) at ambient conditions. In a two-compartment-diffusion cell, a decrease of methanol crossover by 68.6% was observed by introducing SLG sandwiched between two Nafion membranes. The smaller crossover led to an increased open-circuit voltage (OCV) and, thus, to an improved peak power density for highly concentrated fuels. However, they also found a decreased proton conductivity of the SLG-Nafion stack, most pronounced at room temperature. Holmes et al.^5^ investigated SLG in a DMFC setup at elevated temperatures of up to 90 °C. In their study, SLG was transferred onto an amorphous carbon electrode and tested with a Nafion 117 membrane. A significant decrease of methanol crossover by approximately 30% (at 70 °C and 1 M methanol) was observed, accompanied by increased cell performance. The fact that methanol crossover was not completely prevented was explained by defects and cracks in the SLG layer, possibly formed during the transfer process.

Bukola et al.^20^ introduced SLG between two Nafion 211 membranes via a transfer from a copper substrate and investigated the permeability of different cations through this sandwiched SLG composite membrane. It was shown that protons cross over the SLG-Nafion sandwich >100× faster than other investigated cations, which can only pass SLG at defect sites according to their theory. Furthermore, this Nafion-SLG stack was tested in a self-made hydrogen pumping cell setup to evaluate hydrogen crossover and proton conductivity close to room temperature.^18,21^ It was shown that the hydrogen crossover could be reduced 8-fold by introducing SLG between Nafion membranes. Chaturvedi et al.^17^ deployed a similar transfer of SLG from a copper substrate and studied the effect of hot-pressing parameters on the transfer process. They also reported a reduction of hydrogen crossover by around 50% in a custom-built experimental setup at room temperature.

Recently, Kutagulla et al.^19^ compared different two-dimensional materials on top of Nafion 211 with respect to their ability to reduce the hydrogen crossover in operating fuel cells. The barrier layers were additionally protected by a 200 nm thin Nafion coating facing the anode. They report a hydrogen crossover reduction by SLG of around 46% for the first time in a running PEM fuel cell at 80 °C and under full humidification. Nevertheless, they also showed a significant reduction in cell performance (63% lower current density compared to the reference without SLG at 0.6 V) and reported a 35% reduction in proton conductivity of the SLG composite membrane.

These first experimental studies showed promising results of SLG as a barrier interlayer in PEMs for mitigating hydrogen crossover. In general, the hydrogen crossover increases with the temperature, the water content of the membrane, and the hydrogen partial pressure.^22^ As a result of hydrogen crossover, the OCV drops, the efficiency of the cell is impaired and membrane degradation under OCV conditions is accelerated.^6,19,23^ Thus, the implementation of SLG should be beneficial for the overall performance and lifetime of a fuel cell. On the other hand, it was hypothesized that an SLG blocking layer may have a negative impact on the performance of a low-temperature PEM fuel cell (LT-PEMFC) by interfering with water management.^24^ It remains elusive how well SLG withstands repetitive membrane swelling and shrinking from temperature and humidity cycling and the mechanical stress in a standard LT-PEMFC setup under compression, full humidification, osmotic drag, and elevated temperature.

The transfer of two-dimensional (2D) crystals, such as SLG or hexagonal boron nitride, onto membranes by etching from a copper substrate is a commonly used approach.^7,17−20,25,26^ The second transfer method used in this study, based on trivial transfer graphene, which only involves the dissolution of a thin layer of poly(methyl methacrylate) (PMMA) instead of the harsh conditions of metal etching. An equivalent transfer process, but with a self-made PMMA coating on top of SLG and with the removal of the copper substrate before the transfer, was used by Holmes et al.^5^ and also by Chen et al.^24^ to apply SLG on electrodes for DMFC and high-temperature PEMFC MEAs. In the latter study, only a partial diffusion-blocking layer on the electrodes was required to still maintain the essential transport of the electrolyte phosphoric acid into the electrodes.^24^ In contrast to this approach, we aimed for maximum coverage and intactness of the SLG in the PEM to reach the maximum reduction in hydrogen crossover and evaluate all effects resulting from this incorporation.

This work provides a structured screening on the applicability of SLG as a hydrogen-blocking layer in an MEA tested in a commercial 5 cm^2^ LT-PEMFC single-cell setup operating with hydrogen and air at 80 °C and 100% relative humidity (RH). First, the successful manufacturing of the composite membranes obtained from two different SLG transfer methods, namely, a direct transfer from a copper substrate and with trivial transfer graphene, was evaluated using confocal Raman microscopy. To clearly judge the applicability of SLG as a hydrogen-blocking layer in LT-PEMFCs, the ex situ and in situ analysis of the hydrogen barrier function and a detailed analysis of the fuel cell’s performance and water management need to be conducted. Therefore, several diagnostic tools, such as ex situ hydrogen permeation measurements based on mass spectrometry for quantitative hydrogen crossover analysis, in situ electrochemical characterization of the hydrogen crossover, the fuel cell’s performance evaluation supported by impedance and Tafel analysis, and hydrogen pumping experiments for the assessment of the cell’s water management were utilized.

## Experimental Methods

### Materials

Trivial transfer graphene (2.5 cm × 2.5 cm) was purchased from ACS Material LLC, USA. This product consists of a polymeric substrate with chemical vapor deposited (CVD) SLG, which is covered with a thin layer of PMMA. CVD SLG on a copper substrate (2.54 cm × 2.54 cm × 18 μm) was purchased from MSE Supplies LLC, USA. Ammonium persulfate (>98%; abbreviation APS), hydrochloric acid (37%, ACS reagent), ethyl acetate (>99.8%), and 1-propanol (>99.5%) were purchased from Sigma-Aldrich and used without further purification. The Pt/C (TEC10 V40E, Tanaka, Japan) catalyst consisting of Vulcan XC-72 with Pt nanoparticles (40 wt %) with an average diameter of 2.7 nm was used on both electrodes. Nafion XL (NXL) and D2021 Nafion dispersion (both Chemours, USA) were used as membrane and electrode ionomer materials. Glass fiber-reinforced polytetrafluorethylene (PTFE) and virgin PTFE were ordered from Hightechflon, Germany. Pacopads and Kapton were purchased from Pacothane Technologies, USA, and CMC Klebetechnik, Germany, respectively.

### Material Characterization

A confocal Raman microscope (WITec Alpha 300) was used to analyze SLG transferred onto NXL. A 532 nm laser at 25 mW was employed with a water immersion objective (Zeiss 63*x*/1.0 W Plan-Apochromat) to investigate SLG on NXL. Raman spectra of SLG on SiO_2_ and Nafion in ambient conditions were acquired at 5 mW excitation power using a metallurgical objective (Zeiss 100*x*/0.9 EC Epiplan-Neofluar). Spectra were recorded with a Peltier-cooled, back-illuminated EMCCD within a WITec UHTS300 VIS spectrometer with 600 grooves/mm optical grating. Raman spectra were postprocessed by removing cosmic rays and shape-based background subtraction using custom-built MATLAB functions. Raman images are based on sum filters for SLG (G peak: 1550–1650 cm^–1^ and 2D peak: 2600–2750 cm^–1^), Nafion (stretching mode of CF backbone: 700–760 cm^–1^), and water (OH-stretching modes: 3000–3750 cm^–1^). Data from through-plane images were corrected for intensity losses in the *Z*-direction by a linear correction between the summarized intensity of the water-related peak below and above the membranes. Images were taken at a pixel size of 0.5 μm and were scaled up for representation using bicubic interpolation. The intensity distribution of the sum filter images in Figure 2b (see Results and Discussion) was globally adjusted for a clear graphical representation using ImageJ (raw sum filter images are displayed in Figure S1 in the Supporting Information). Samples analyzed by Raman microscopy were solely used for this characterization method and not processed or analyzed further.

Cross-sections of MEAs were imaged by scanning electron microscopy (SEM) using a Tescan Vega 3 with a secondary electron detector. The cross-section was prepared by ultramicrotomy using a DiATOME diamond knife in an RMC Boeckeler PowerTome after embedding the sample in Araldite 502 epoxy resin. The prepared cross-sections were sputter-coated with gold (Cressington 108 manual) before imaging to avoid charging artifacts.

### Membrane Fabrication

**Figure 1:**
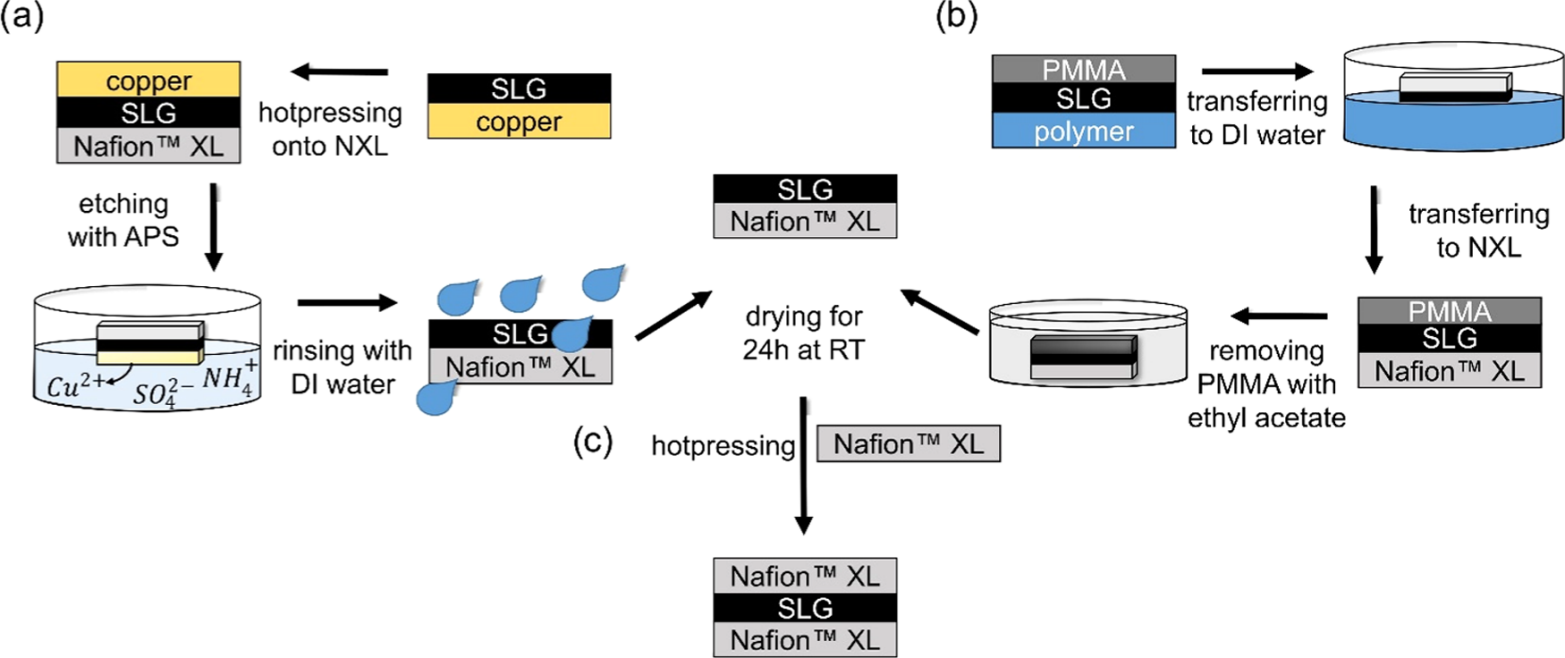
Scheme of the two different transfer methods of SLG. (a) The transfer method for SLG (CT) based on SLG deposited on a copper substrate. (b) The transfer process for SLG (TT) based on trivial transfer graphene. (c) Hot pressing the SLG-NXL membrane with a second NXL to form an NXL-SLG-NXL composite membrane.

A stack of two NXL membranes (dry thickness of 27.5 μm each) without an SLG interlayer was used as a reference system. We employed the reinforced membrane NXL, as it was found by Shi et al.^27^ that the in-plane swelling of this membrane is lower than for standard perfluorosulfonic acid (PFSA) membranes like Nafion 212. Two different transfer routes, depicted in Figure 1, were employed to incorporate the SLG into the membrane stack. SLG on a copper substrate (Figure 1a, which will be referred to as SLG (CT)) was transferred onto NXL as described in the literature.^17−20,25,26^ In brief, CVD SLG (CT) was hot-pressed onto an NXL membrane (150 °C, 2.5 MPa, 7 min), with the SLG facing the membrane, in between two glass fiber-reinforced PTFE sheets with Pacopads, followed by etching of copper with 50 mL of a freshly prepared 0.26 M APS ((NH_4_)_2_S_2_O_8_)-solution for 1 h, which removed all visible remnants of copper according to the following reaction (eq 1)^28^
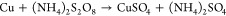
1Taking the molar amount of the copper substrate and the APS solution into account, there is an excess of APS with a factor of 8 compared to copper (see the Supporting Information). Equation 2 shows the reaction of persulfate ions with water, which is known to occur in neutral and dilute acid solutions.^29^
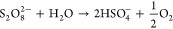
2After this process, NXL with SLG (CT) was thoroughly rinsed with DI water (at least 15 times) at room temperature (RT) to remove the remaining etching solution and copper ions. The composite membrane was then dried at RT for 24 h.

The second transfer route is based on trivial transfer graphene (Figure 1b, which will be referred to as SLG (TT)), which consists of SLG coated with PMMA and deposited on a polymeric substrate. The trivial transfer graphene with PMMA coating was transferred according to the instructions from ACS Material LLC. The SLG coated with PMMA was transferred to a bath of DI water and left floating for 2 h. Afterward, the SLG with the PMMA coating was transferred onto an NXL membrane by lifting the membrane from below the water surface and collecting the SLG with PMMA coating on top of the membrane. The resulting composite membrane was first dried at RT for 1 h, then additionally dried at 80 °C for 20 min. Finally, the PMMA coating of the SLG was removed by immersing the composite membrane in 50 mL of ethyl acetate for 1 min. The membrane was dried at RT for 24 h prior to further use. Photographs of both transfer routes are provided in Figure S2 (Supporting Information). Finally, for both transfer routes, a second NXL membrane was hot-pressed (155 °C, 2.5 MPa, 5 min) onto the NXL-SLG composite membrane in between two glass fiber-reinforced PTFE sheets and Pacopads, eventually resulting in an NXL-SLG-NXL stack with the SLG in the center (Figure 1c). For analyzing the effect of the APS etching on the MEA performance, an additional APS reference membrane, which was treated exactly like the SLG (CT) composite membrane but without the actual SLG transfer, was manufactured. Therefore, an NXL membrane was hot-pressed as described for the first SLG (CT) step and soaked in APS for 1 h. Further, it was thoroughly (at least 15 times) rinsed with DI water at RT to remove the remaining APS, dried for 24 h, and finally hot-pressed (155 °C, 2.5 MPa, 5 min) with a second NXL membrane.

### Decal Electrodes and MEA Assembly

Decal electrodes were fabricated from a commercial Pt/C (TEC10 V40E) catalyst, an ionomer dispersion (Nafion D2021), water, and 1-propanol. The solid content of the ink was 13.5 wt %, the *I*/*C* ratio was 0.65, and the water content of the solvent mixture was 10 wt %. ZrO_2_ beads were added to the ink as a grinding medium, and the ink was mixed for 24 h at 60 rpm on a roller mill. The Mayer rod technique was used to coat the ink onto a 50 μm virgin PTFE substrate at a wet film thickness of 100 μm. The electrodes were dried at RT and subsequently at 60 °C for 1 h, cut into 5 cm^2^ pieces, and transferred to membranes via the decal process (155 °C, 2.5 MPa, 7 min of hot pressing, see composite membrane fabrication) to form catalyst-coated membranes (CCMs). The electrode loading was determined as the weight difference of the decals with and without the catalyst layer. MEAs tested in fuel cell setups had anode Pt loadings of 0.15 ± 0.03 mg_Pt_ cm^–2^ and cathode Pt loadings of 0.28 ± 0.01 mg_Pt_ cm^–2^. For hydrogen pumping experiments, the Pt loadings were 0.3 ± 0.01 mg_Pt_ cm^–2^ for working and reference electrodes.

MEAs were assembled using H23C8 gas diffusion layers (GDLs) from Freudenberg. The cell fixtures were tightened with 5 N m torque, and the compression of the GDLs was adjusted with glass fiber-reinforced PTFE gaskets to 30%. If not specified otherwise, two independent measurements were conducted each to ensure the reproducibility of the obtained results. The average values of those two measurements are displayed with error bars based on the absolute deviation.

### PEMFC Performance Tests and MEA Characterization

PEMFC tests were performed using a Scribner 850e fuel cell system combined with a backpressure unit and a Scribner 885 potentiostat or a VSP-300 potentiostat from BioLogic SAS. Scribner cell fixtures, including aluminum end plates, gold-plated copper current collectors, and serpentine-patterned single-channel graphite flow fields with an active cell area of 5 cm^2^, were used. Linear sweep voltammetry (LSV) measurements and impedance measurements with H_2_/N_2_ were conducted with a VSP-300 from BioLogic SAS.

PEMFC performance tests were conducted at 80 °C cell temperature with fixed flow rates of 0.25 l min^–1^ H_2_ at the anode and 0.75 l min^–1^ synthetic air at the cathode (21% vol O_2_ in N_2_), under fully humidified conditions and at 1.5 bar_abs_. No differential pressure operation was employed. The cell was conditioned to 80 °C, 100% RH, and 1.5 bar_abs_ for 1 h with 1 l min^–1^ H_2_ and 1 l min^–1^ N_2_. The break-in of all MEAs consisted of a constant potential hold at 0.6 V for 25 min, 5 min at OCV, and 5 min at 0.3 V, repeated three times. After break-in, galvanostatic polarization curves in the range of 0–1600 mA cm^–2^ with a hold time of 60 s at all current densities and a potential limit of 0.2 V were performed. Impedance measurements were performed in a frequency range from 10 kHz to 10 Hz at 25 mA cm^–2^ (for the first set of MEAs) or 20 mA cm^–2^ (for the second set of MEAs) and 600 mA cm^–2^ (for all MEAs) under the same conditions. LSV scans after the break-in and performance analysis of the MEAs were performed under H_2_ (reference electrode) and N_2_ (working electrode) gas supply with a constant flow of both gases of 0.2 l min^–1^, with a scan rate of 2 mV s^–1^ at 1.5 and 2 bar_abs_. LSV measurements were repeated three times with 10 min OCV hold between each measurement and averaged afterward. Additional LSV scans executed at 30 °C before cell conditioning were inconclusive, while those recorded at 80 °C can be found in the Supporting Information (Figure S3). There, we investigated the crossover behavior by LSVs prior to the cells’ break-in and performance test to avoid possible damage to the SLG interlayer during these processes. The MEAs showed a constant increase in current density during the LSV measurement, which can be related to their pristine state prior to the break-in, while the relative reduction in hydrogen crossover was comparable to the LSV scans after the break-in. High-frequency resistance (HFR) corrected polarization curves were obtained after an HFR correction based on eq 3.
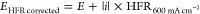
3The HFR values (HFR_600 mA cm^–2^_) were determined as the intercepts with the real axis in the Nyquist plots of the impedance spectra recorded at 600 mA cm^–2^.

Tafel plots of the polarization curves were corrected for the hydrogen crossover rate as determined from LSV measurements (*i*_H_2_ crossover_), the HFR in the activation region recorded at 20/25 mA cm^–2^ (HFR_20/25 mA cm^–2^_), and the effective proton sheet resistance of the cathode (R_H^+^, cath_^eff^) as described in eqs 4–6.^30^ Since the LSV measurements showed no significant electric shorts for the membranes, no correction for electric shorts was performed.
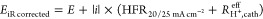
4with
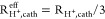
5
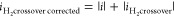
6The proton sheet resistance (R_H^+^, cath_) was evaluated from impedance measurements (frequency range from 100 kHz to 10 Hz) acquired under H_2_/N_2_ conditions at OCV and at 1.5 bar_abs_, 100% RH, and a constant flow of 0.2 l min^–1^ of the gases. It was determined based on the transmission line model.^30−33^ The acquired data were evaluated with the “Z-fit” impedance fitting tool of the EC-lab software from BioLogic SAS. An equivalent circuit with a resistor R for the HFR, a restricted linear diffusion element *M*_a_ for the proton sheet resistance, and an inductor *L*_a_ for the inductance of the setup was used to determine the proton sheet resistance. Since the catalyst utilization is above 90% in the Tafel region, the effective proton sheet resistance of the cathode (R_H^+^, cath_^eff^) was calculated using eq 5.^30^ Please note that no *iR*-correction was performed for the full polarization data due to the catalyst poisoning observed for the SLG (CT) and the possible copper contamination of the ionomer in the cathode catalyst layer, which can affect the catalyst utilization and therewith the effective proton sheet resistance.

### Hydrogen Pumping

Hydrogen pumping was performed to investigate the effect of the SLG interlayer on the water management of the membranes. The cells were conditioned at 80 °C for 2 h with 0.25 l min^–1^ H_2_ at working and reference electrodes with a pressure of 1.5 bar_abs_, an active area of 5 cm^2^, and Pt loadings of 0.3 ± 0.01 mg_Pt_ cm^–2^ on each electrode. The gas stream at the working electrode (hydrogen oxidation reaction; HOR) was humidified to 100% RH and at the reference electrode (hydrogen evolution reaction; HER) to 50 or 100% RH, as indicated in the description of the results. A galvanostatic experiment from 0.4 to 1.8 A cm^–2^ in 0.2 A cm^–2^ steps was performed, which was terminated when exceeding a cell voltage of 1 V. Each current density was held for 30 min, and the average potential and standard deviation of the last 60 s was evaluated.

### Hydrogen Permeation

Permeation measurements via gas composition analysis were executed on a dedicated test stand connected to a Thermo Scientific Prima BT mass spectrometer (MS, 1 kV ion source, scanning magnetic sector analyzer, Faraday detector) for exhaust gas analysis. A custom-made single cell with a 5 cm^2^ active area and a single-channel serpentine flow field was used. The as-prepared composite membranes were directly sandwiched between two H23C8 GDLs. Gaskets were chosen to achieve a nominal compression of 10%. MEA samples after fuel cell testing were tested by transferring the full stack (MEA, GDLs, and gaskets) to the permeation measurement setup. All measurements were conducted at 80 °C cell temperature using either dry or fully humidified gas streams and with symmetric backpressure on both sides of the sample. The reference side was flushed with 50 sccm of H_2_, and the analysis side with 200 sccm of N_2_. All samples were analyzed after full dry-out, under fully humidified conditions, and again after dry-out. To achieve equilibrium, the samples rested for at least 12 h in the dry or humidified gas streams before permeation rates were recorded. In order to determine the permeation coefficient of hydrogen through the different samples in their dry and wet states, pressure steps of 1, 1.1, 1.3, 1.5, 2, 3, and 4 bar_abs_ with respect to the partial pressure of hydrogen on the reference side were set for at least 10 min each. The applied backpressure for measurements with humidified gas streams was corrected for the partial pressure of water at 80 °C (i.e., + 0.474 bar). After equilibration of the MS hydrogen signal in the sample stream, at least the last 1 min was averaged to get the permeation rate at each pressure step. Plotting the permeation rates versus the pressure difference of hydrogen over the membrane, a line through the origin can be fitted for a purely diffusion-limited process, whereas the slope represents the hydrogen permeability coefficient of the sample under the respective conditions. In agreement with the literature,^34^ the permeability coefficient extracted from the linear fit is referenced to the dry thickness of the membrane.

## Results and Discussion

Transferring SLG onto a PEM can be performed using different transfer methods. We employed CVD SLG deposited on a copper substrate and trivial transfer graphene to fabricate composite membranes with an SLG interlayer. For both transfer procedures, schematic overviews and details about the transfer processes are provided in the Experimental Section (Figure 1) and the Supporting Information (Figure S2). In the following sections, the transfer method via SLG on a copper substrate is abbreviated as SLG (CT), and the process, including trivial transfer graphene, is referred to as SLG (TT).

**Figure 2:**
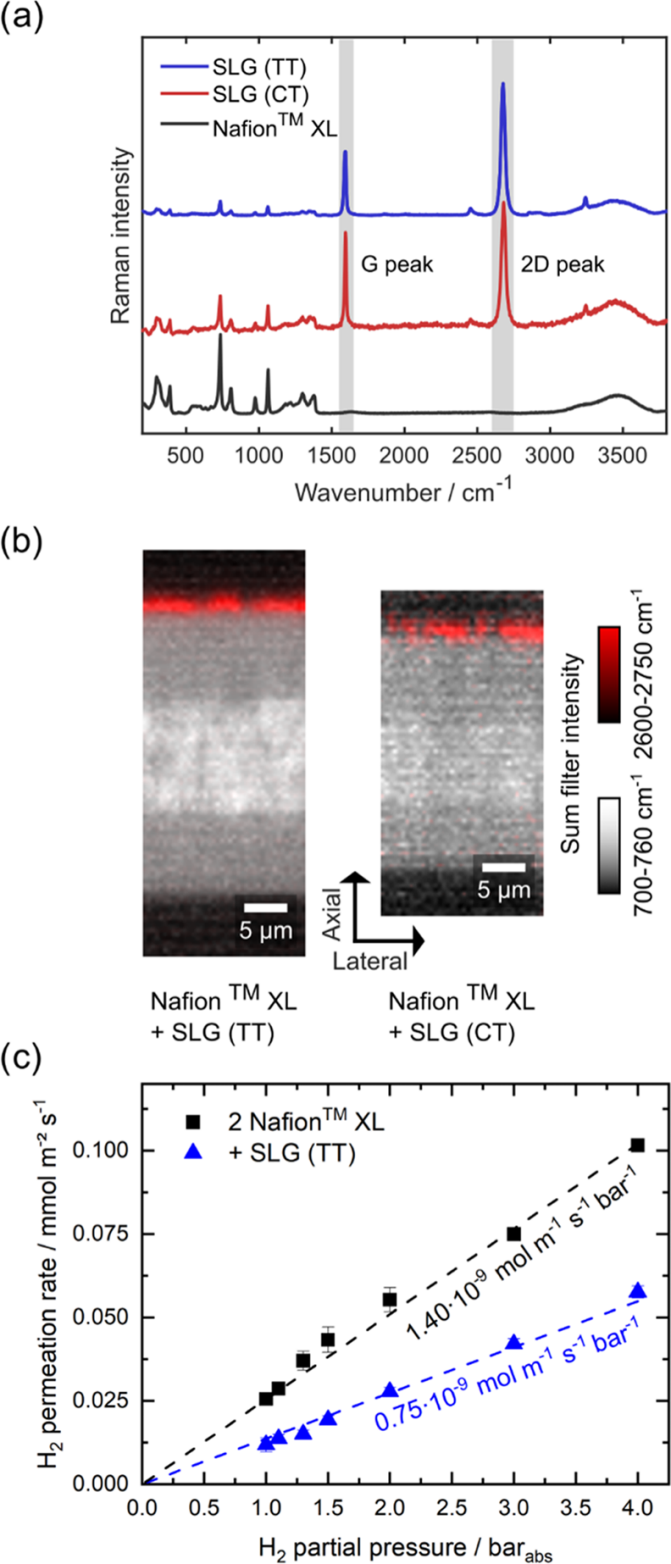
Composite membrane analysis. (a) Raman spectra of SLG transferred via the trivial transfer graphene process (SLG (TT); blue line) or the transfer from a copper substrate (SLG (CT); red line) and a spectrum of Nafion XL (NXL) as a reference (black line). (b) Raman through-plane scans of SLG-NXL composite membranes. Sum-filter-based Raman images are displayed for the CF backbone stretch (white) and the 2D SLG peak (red; SLG (TT) on the left and SLG (CT) on the right). All samples were analyzed in DI water, and the intensity distribution of the sum filter images was adjusted globally for each sum filter for graphical representation. See Figure S1 for unprocessed sum filter images of the hyperspectral image scans. (c) Hydrogen permeation rates at 80 °C, 0% RH, measured at different pressures for two hot-pressed NXL membranes (reference, black squares) and the SLG (TT) composite membrane (blue triangles). Numbers represent the permeation constants extracted from the linear fit (dashed black or blue line) referenced to the dry nominal thickness of 55 μm for both membrane types.

The transfer of SLG onto NXL membranes was confirmed using confocal Raman microscopy. Spectra of SLG were visible on top of the membranes (Figures 2a,b and S1), with additional signals from the ionomer due to the diffraction-limited spatial resolution of the microscope. All samples show the typical C–F stretching mode of the PTFE backbone at approximately 730 cm^–1^, as described in the literature for Nafion.^35^ Also, the broad OH-stretching mode at around 3500 cm^–1^ can be observed in all samples, resulting from the hydration of the samples since they were investigated in water immersion. SLG can be identified by intense G and 2D peaks at around 1590 and 2685 cm^–1^.^25,36^ We found that on the one hand, the ratio between G and 2D bands is not equal for SLG (CT and TT) composite membranes, but on the other hand, that this ratio is also sensitive to adjacent materials and presumably defects, and can therefore be explained by local effects (compare Supporting Information and Figure S4). The presence of a D band at about 1350 cm^–1^ indicates defects in SLG.^25,36,37^ Here, for SLG (CT and TT) composite membranes, a weak peak can be observed at this spectral position, which is convoluted with the Raman spectrum of Nafion. The occurrence of a D band in the spectra of SLG after the transfer onto, e.g., a silicon wafer or a membrane was also observed in other studies.^10,20,25^Figure 2b (compare Figure S1 for unprocessed scans) shows through-plane Raman images of SLG (CT and TT)-NXL. Additionally, we performed in-plane Raman mappings of SLG (TT) on quartz slides and on NXL at multiple positions to assess the quality and coverage of the monolayer (Figures S5–S10). The results indicate a complete transfer of SLG without any local loss of SLG, at least within the scale of the resolution limit of confocal microscopy. At the same time, the occurrence of D peaks suggests local defects in the SLG.

Both SLG (TT) and SLG (CT) on NXL show intensity variations in sum-filter-based images for the 2D (Figure 2b) and G peaks of SLG (Figure S1), which can be explained by defects in the monolayer and local variations in the interfacing between SLG and the ionomer membranes. The thickness difference between the ionomer membranes of SLG (TT) on NXL and SLG (CT) on NXL in Figure 2b is caused by the different transfer routes, as the latter involves a hot-pressing step at a temperature exceeding the membrane’s glass transition temperature (compare Figure S1).

After confirming the successful transfer of SLG to the NXL membrane and the full assembly of the NXL-SLG-NXL composite membranes, the hydrogen-blocking capability of these composite membranes in comparison to the pure double NXL reference was evaluated by permeation measurements at 80 °C and 0% RH as explained in the Experimental Methods. The results for the double NXL reference (black) and the SLG (TT) composite membrane (blue) are shown in Figure 2c.

With increasing partial pressure of hydrogen, its permeation rate increases linearly, as expected for a diffusion-limited process as gas permeation through PFSAs. This behavior can be observed for both samples. Notably, the permeation rate for the SLG composite membrane is roughly half of the double NXL reference membrane. By employing a linear fit to the pressure-dependent permeation rates, one can estimate the permeation constants of hydrogen through the different membranes, which are depicted by the dashed lines in Figure 2c, resulting in hydrogen permeabilities of 1.40 × 10^–9^ mol m^–1^ s^–1^ bar^–1^ for the double NXL reference and 0.75 × 10^–9^ mol m^–1^ s^–1^ bar^–1^ for the SLG-containing membrane. The permeability of the reference is well in the range of literature values,^22,38^ while the permeability of the composite membrane is significantly reduced by 46% at dry conditions.

**Figure 3:**
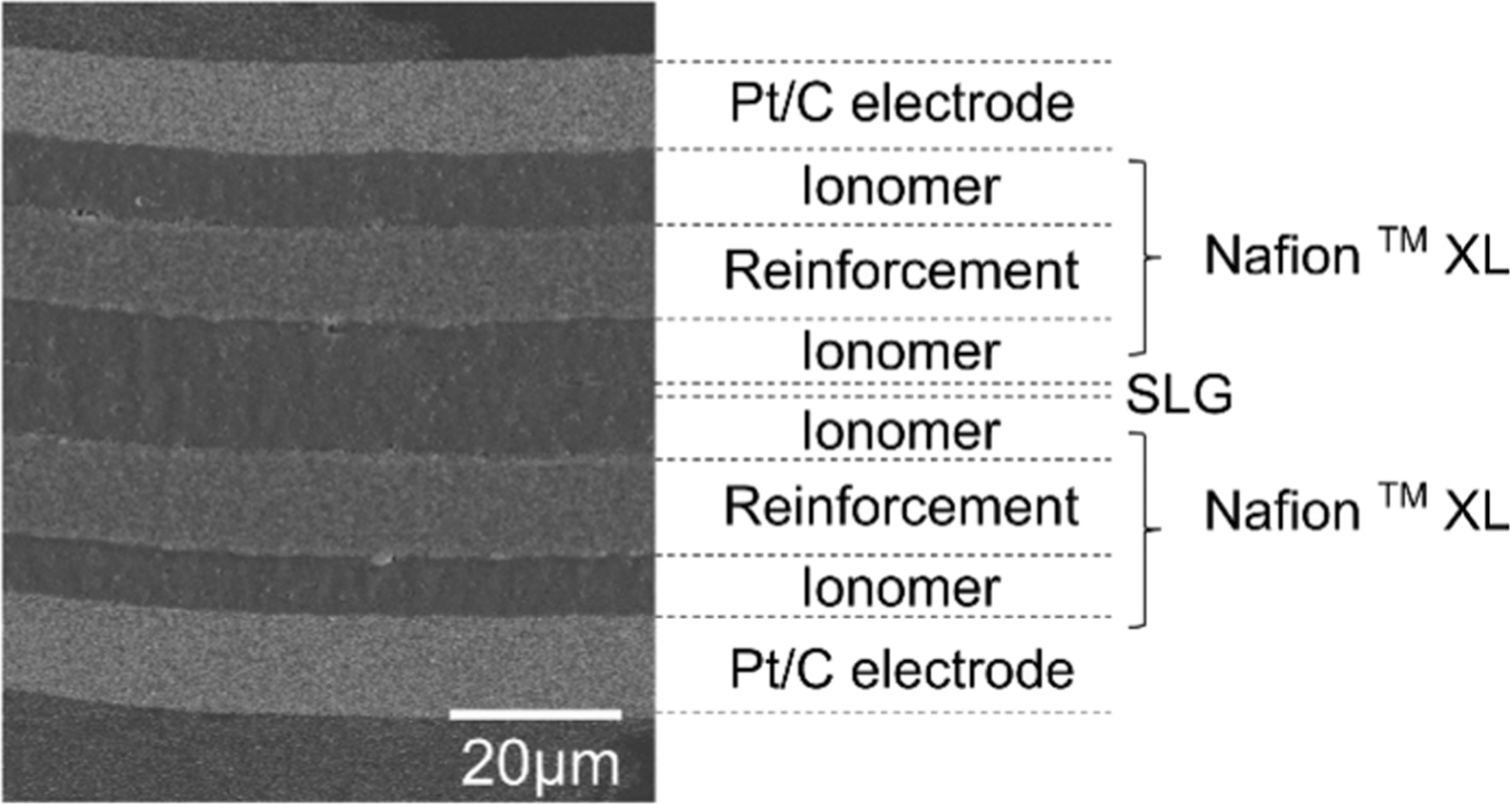
Exemplary cross-sectional SEM image of an MEA with the SLG (TT) composite membrane after hydrogen pumping. The thickness of the composite membrane was determined to be 54.5 ± 0.69 μm (Figure S11 in the Supporting Information), in good agreement with the nominal thickness of the dry double NXL membrane of 55 μm.

**Figure 4:**
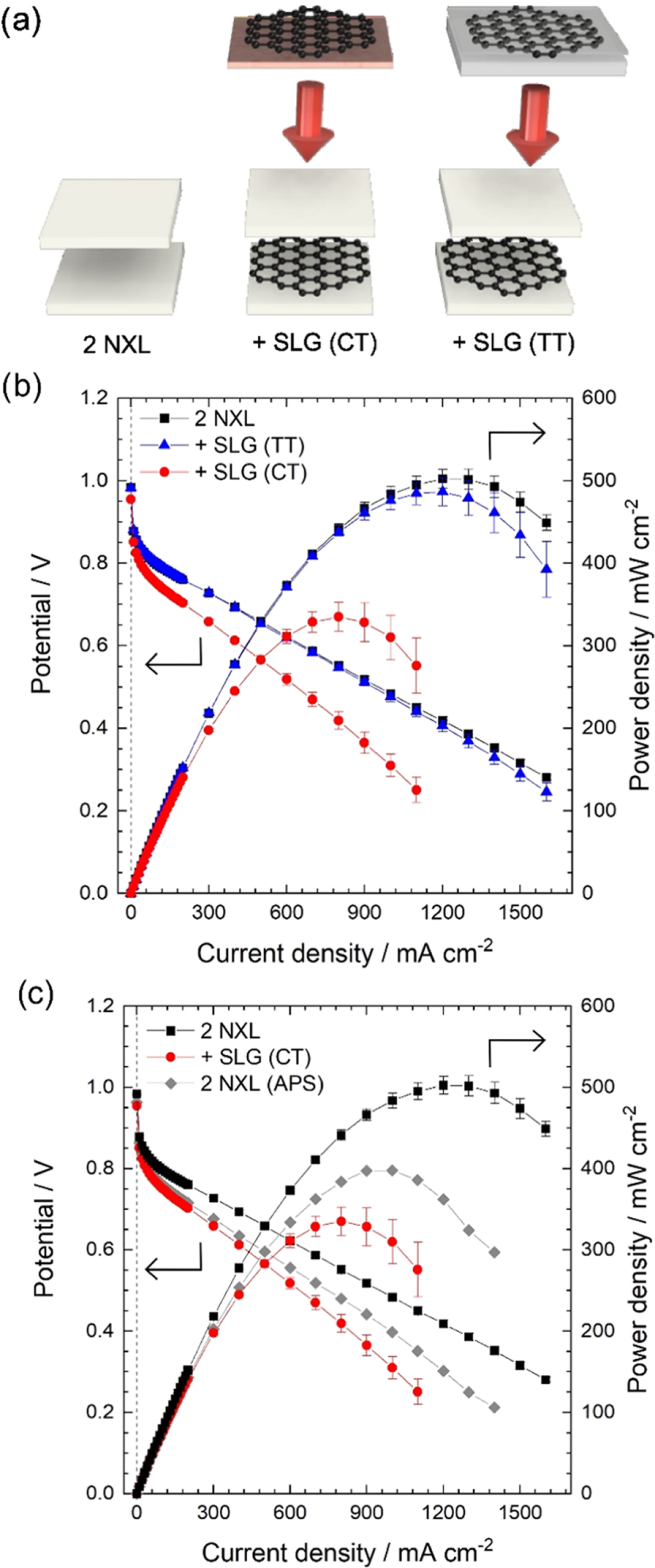
(a) Schematic overview of the structure of the double NXL reference, SLG (CT) and SLG (TT) composite membranes. (b) Fuel cell performance analysis of MEAs assembled with the double NXL reference membrane (black squares), the SLG (TT) (blue triangles), and the SLG (CT) composite membranes (red circles). (c) Fuel cell performance analysis of the double NXL reference MEAs (black squares) in comparison to an MEA assembled with a reference membrane treated with ammonium persulfate (APS) (gray diamonds) and the MEAs assembled with the SLG (CT) membrane (red circles). In (b, c), a line was added between data points to guide the eye. All fuel cell tests were performed at 80 °C, 100% RH, 1.5 bar_abs_, H_2_ flow of 0.25 l min^–1,^ and airflow of 0.75 l min^–1^. Displayed are the mean values of two independently measured MEAs for each membrane type (except for the double NXL reference treated with APS, which was done once). Error bars indicate the absolute deviation from the mean value.

Single-cell hydrogen/air fuel cell tests at standard LT-PEMFC conditions of 80 °C and 100% RH were performed to analyze the effect of the SLG layer within the PEM on the electrochemical performance. A cross-section of an MEA can be seen in Figure 3, representing the assembly of all measured MEAs. Sketches of the three investigated composite membrane types (double NXL reference, SLG (TT), and SLG (CT)) are depicted in Figure 4a. Additionally, the cross-sections of the tested MEAs after fuel cell testing are depicted in Figure S12 in the Supporting Information. A thickness evaluation of all three membrane types (Figure S13 in the Supporting Information) confirms the unity in thickness of all tested membranes. Figure 4b shows the polarization data and power densities of the analyzed MEAs. The key numbers of the polarization analysis (OCV, peak power density, HFR, and proton conductivity) are further summarized in Table 1.

**Table 1:** Summary of OCVs, Peak Power Densities, and HFRs at 600 mA cm^–2^ during Performance Tests of the Double NXL Reference MEAs, SLG (TT) MEAs, SLG (CT) MEAs, and an APS-Treated Double NXL Reference MEA (results are provided in Figure 4b,c)^a^

membrane	OCV (mV)	peak power density (mW cm^–2^)	HFR at 600 mA cm^–2^ (mΩ cm^2^)	proton conductivity (mS cm^–1^)
2 NXL	984 ± 3	502 ± 11	101 ± 3	61 ± 2
SLG (TT)	982 ± 4	486 ± 17	113 ± 5	54 ± 2
SLG (CT)	955 ± 1	335 ± 17	133 ± 8	45 ± 3
2 NXL (APS)	963	398	109	56

a All fuel cell tests were performed at 80 °C, 100% RH, 1.5 bar_abs_, H_2_ flow of 0.25 l min^–1^, and airflow of 0.75 l min^–1^. Stated are the mean values ± absolute deviation of two independent MEAs for each membrane type (except for the double NXL reference treated with APS, which was only done once). The proton conductivity of each membrane type was calculated from the HFR value measured in the fuel cell experiments at 600 mA cm^–2^ under consideration of the electrical resistance of the fuel cell setup of 11 mΩ cm^2^ (full assembly, excluding CCM) and with the nominal dry thickness of the membranes of 55 μm.

The MEAs of the double NXL reference membrane (black squares) and the SLG (TT) composite membrane (blue triangles) show similar polarization data with a maximum power density of around 500 mW cm^–2^ and only a slight reduction in the membrane conductivity (Table 1). This finding is in good agreement with the work of Bukola et al.,^25^ who measured 34 mΩ cm^2^ as the contribution of SLG to the HFR of a PFSA membrane at 30 °C. Chaturvedi et al.^17^ detected a contribution of SLG to the area-specific proton resistance of 50 mΩ cm^2^ at room temperature. Since the conductivity of SLG increases with temperature,^16^ a 12 mΩ cm^2^ higher HFR for SLG (TT) at 80 °C compared to the double NXL reference MEA (113 ± 5 versus 101 ± 3 mΩ cm^2^) is in the expected range (see Table 1). Therefore, the overall performance of the MEA was not impaired significantly by the implementation of SLG (TT).

In contrast, the maximum power density of the MEA with the SLG (CT) composite membrane (red circles) was reduced drastically by 33% compared to the double NXL reference MEA, which is caused by increased activation polarization and ohmic losses. Minding that the transfer via both SLG (CT) and SLG (TT) methods led to similar structures of the composite according to the Raman analysis (see Figures 2 and S1), the question remains as to why the composite membrane with SLG (CT) shows such a dramatic performance reduction.

As clearly visible from the raw polarization data in Figure 4b, the MEAs made from SLG (CT) membranes show increased ohmic polarization, which is reflected in the 18% higher HFR compared with the SLG (TT) MEA and in the 32% higher HFR compared with the double NXL reference MEA (see Table 1). The increase in the HFR for the SLG (CT) MEA is still lower than the increase in the HFR of 61% presented in the study of Kutagulla et al.^19^ for an SLG-N211 composite membrane, where SLG was also transferred via a direct etching process with APS and tested under the same conditions. The authors reported a decrease in peak power density by 46 and a 63% lower current density at 0.6 V with the implementation of SLG directly transferred from a copper substrate. Thus, their study confirms the negative effect of SLG (CT) on fuel cell performance, though the extent of performance reduction is lower in our case (33% peak power density reduction), probably due to the slight differences in the etching procedure.

A possible explanation for the reduced conductivity of the MEA assembled with the SLG (CT) composite membrane is remnant ammonium ions (NH_4_^+^) from the etching solution APS that are not entirely removed from the membrane. Positively charged ions can interact with the anions of the sulfonic acid groups within the membrane and diminish its proton conductivity.^39,40^

Therefore, the impact of the etchant on the membrane was investigated. An NXL membrane was treated equally to the copper transfer approach but without actually transferring SLG from the copper substrate and hot-pressed with a nontreated second NXL membrane (see Experimental Methods for details). The APS treatment indeed resulted in a peak power density reduction of 104 mW cm^–2^ (equals 21% loss) compared to the double NXL reference while still outperforming the SLG (CT) composite membrane (gray diamonds, Figure 4c). Furthermore, the HFR in the ohmic region (600 mA cm^–2^) of the APS-treated MEA was only slightly increased compared to the double NXL reference and similar to the SLG (TT) MEAs (Table 1), indicating that the negative effect of the etchant without copper to be oxidized is not affecting the membrane’s conductivity severely. However, the APS-treated MEA showed an increased activation polarization and decreased OCV.

If the etchant solution alone is not responsible for the reduced proton conductivity of the membrane, the combination of etchant and copper will be responsible for the impaired performance of the SLG (CT) MEA. Copper ions are known to reduce the ionic conductivity of Nafion and, therefore, also the performance of PEMFCs.^40,41^ To investigate the presence of copper ion remnants in the composite membrane after the direct transfer of SLG from a copper substrate, samples of the SLG (CT) composite membrane and a reference were analyzed by inductively coupled plasma mass spectrometry (ICP-MS) to detect the presence of copper ions in the membrane (see the Supporting Information for information on the procedure and an estimation of the contamination of the membrane). The analysis revealed the presence of copper ions in the SLG (CT) membrane, whereas no copper ions were found in the reference (Figure S14). Thus, it can be concluded that copper ion remnants likely occupy sulfonic acid groups in the PFSA, impairing its proton conductivity and thereby increasing the HFR (Table 1).

The reduced ionic conductivity of the SLG (CT) composite membrane can be explained by the occupation of sulfonic acid groups with copper and ammonium cations, but the increased activation polarization and decreased OCV hint toward additional catalyst poisoning.

**Figure 5:**
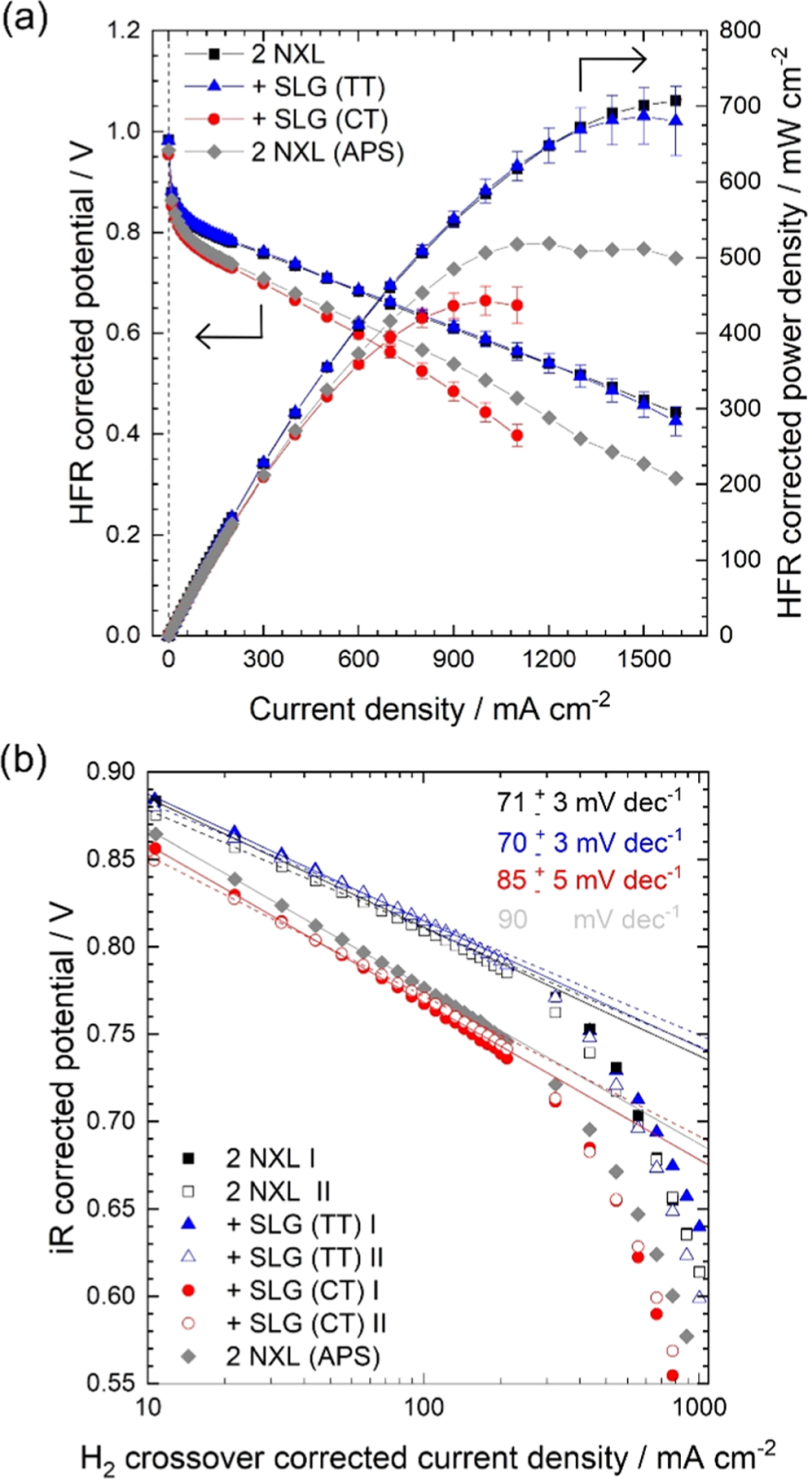
(a) Summary of HFR-corrected fuel cell tests including the double NXL reference MEA (black squares), the MEA assembled with the APS-treated double NXL reference membrane (reference (APS); gray diamonds), and the MEAs assembled with SLG (TT) (blue triangles) or SLG (CT) (red circles). Displayed are the mean values of two independently measured MEAs for each membrane type (except for the double NXL reference treated with APS, which was done once); error bars indicate the absolute deviation of the mean value. A line was added between data points to guide the eye. (b) Tafel plots of all tested MEAs drawn from the polarization curves after correction for hydrogen crossover, HFR, and proton sheet resistance. All fuel cell tests were performed at 80 °C, 100% RH, 1.5 bar_abs_ pressure, H_2_ flow of 0.25 l min^–1^, and airflow of 0.75 l min^–1^. A linear fit was applied for the data points below 100 mA cm^–2^ to determine the Tafel slopes.

The HFR-free polarization curves (Figure 5a) reveal a similarly reduced potential in the activation region for the SLG (CT) MEA and the APS-treated MEA compared with the double NXL reference and the equally performing SLG (TT) MEA (blue triangles). With increasing current density, the SLG (CT) MEA still shows larger losses than the APS-treated double NXL reference, which could be due to the fact that the HFR correction only accounts for the membrane conductivity, whereas the ionomer in the electrodes can be equally affected, which results in ohmic losses that may not be covered fully by the HFR. The potential loss in the activation region of the SLG (CT) MEA and the APS-treated MEA hints toward catalyst poisoning by the etchant solution, which was evaluated by a Tafel analysis of the fuel cells (Figure 5b). Both the APS-treated double NXL reference and the SLG (CT) MEA show an increase in the Tafel slope of 85 ± 5 mV dec^–1^ for the SLG (CT) MEAs and 90 mV dec^–1^ for the APS-treated double NXL reference, compared to the Tafel slopes of the double NXL reference and SLG (TT) MEAs of 71 ± 3 and 70 ± 3 mV dec^–1^, respectively. A possible explanation for the higher losses in the kinetic region is an interaction between SO_4_^2–^ anions formed during the etching step with APS (eq 2) and active Pt sites. Kabasawa et al.^42^ showed that introducing 50 mM H_2_SO_4_ in a running PEMFC experiment at 80 °C resulted in a drop of cell voltage by 23 mV and an increase in Tafel slope by around 8 mV dec^–1^. This effect was attributed to the adsorption of sulfate, which reduced the number of active sites for the oxygen reduction reaction. The reduction in OCV of ∼30 mV for the SLG (CT) cell and ∼20 mV for the APS-treated double NXL reference can also be attributed to sulfate poisoning (see eq 2). Thus, the performance losses of the SLG (CT) fuel cell can be traced back to a combination of reduced catalytic performance due to the APS treatment and an increased proton conduction resistance due to remnants of copper and ammonium ions within the composite membrane. Since the MEA incorporating SLG (TT) shows a similar OCV, peak power density, and HFR compared to the double NXL reference, we conclude that SLG does not impair the fuel cell performance.

There are treatments available for cleaning and regenerating PFSA membranes, such as boiling in H_2_SO_4_ and DI water, which could be employed on SLG (CT) composite membranes to avoid the detrimental effects of the transfer process on the performance.^41^ However, these additional steps might result in the introduction of additional defects to the SLG; therefore, the trivial transfer graphene process appears as the more viable route. Thus, we omitted further experiments on additional cleaning steps for SLG (CT) but focused on SLG (TT) instead. It shall be noted that the use of SLG directly etched from copper on cation exchange polymers requires intensive cleaning procedures to eliminate remnant copper cations and contaminations from the etching solution. Holmes et al.^5^ and Chen et al.^24^ presented a transfer equal to trivial transfer graphene, where they transferred graphene from a copper substrate onto a thin film of PMMA as an intermediate step and then finally transferred graphene from this PMMA layer onto fuel cell electrodes to avoid the direct contact between copper, copper etchant, and MEA components. Hence, it can be concluded that incorporating SLG into the PEM does not negatively affect the performance of the resulting fuel cell, while the transfer process has to be evaluated critically.

**Figure 6:**
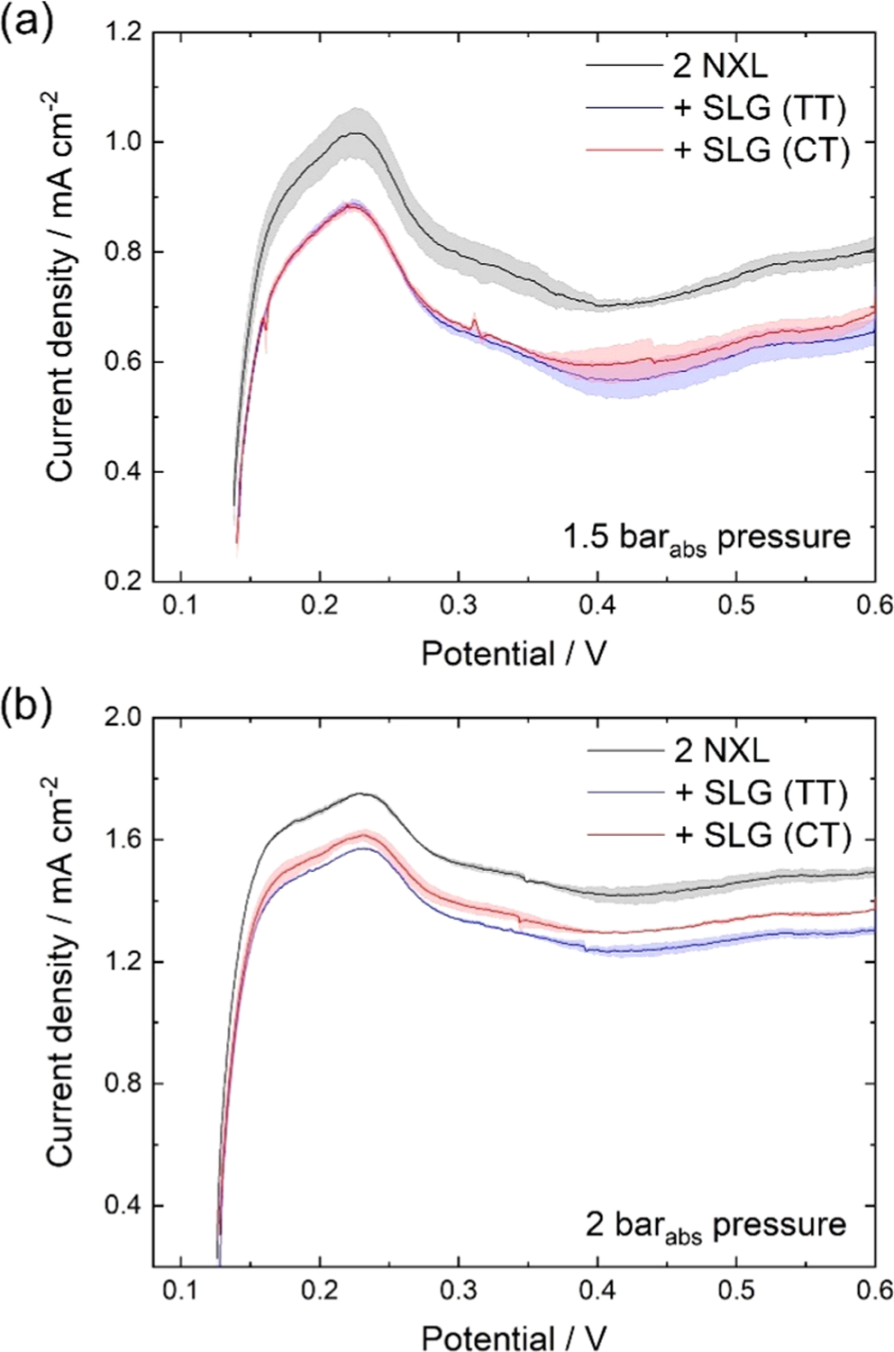
Linear sweep voltammetry (LSV) of the MEAs after the break-in and performance test. LSV measurements were conducted at 0.2 l min^–1^ H_2_ at the fuel cell anode compartment and 0.2 l min^–1^ N_2_ at the fuel cell cathode compartment at 80 °C, 100% RH, and a scan rate of 2 mV s^–1^. Represented are the mean values with the absolute deviation indicated by the shaded area of two independently measured MEAs each. (a) LSV scans at 1.5 bar_abs_ (symmetric) and (b) LSV scans at 2 bar_abs_ (symmetric).

The hydrogen crossover of the fuel cells was determined electrochemically by LSV since this technique is broadly accepted and practiced in the literature^6,9,43,44^ (Figure 6). It decreased by 15–19% in the cells with SLG compared to the double NXL reference MEA at 1.5 bar_abs_ and by 9–13% at 2 bar_abs_, calculated for the mean crossover current density at 0.4 V. Permeability coefficients extracted from the LSVs are reported in Tables 2 and S1.

**Table 2:** Hydrogen Permeabilities of the 2 NXL Reference and the SLG (TT) Composite Membranes at 80°C Measured via MS (Dry and Wet) and Estimated from the LSV Data as Shown in Figure 6^a^

sample	membrane	measurement conditions		ε_H_2__ (10^–9^ mol m^–1^ s^–1^ bar^–1^)
as prepared, MS permeability	2 NXL	dry	before humidification	1.40 ± 0.02
			after humidification	1.37 ± 0.04
		100% RH streams		3.13 ± 0.04
	+SLG (TT)	dry	before humidification	0.75 ± 0.02
			after humidification	0.65 ± 0.05
		100% RH streams		2.46 ± 0.05
after FC test, MS permeability	2 NXL	dry	before humidification	1.38 ± 0.04
			after humidification	1.32 ± 0.03
		100% RH streams		3.13 ± 0.05
	+SLG (TT)	dry	before humidification	1.06 ± 0.07
			after humidification	1.10 ± 0.06
		100% RH streams		2.83 ± 0.06
FC test, LSV permeability	2 NXL	100% RH, 1.5 bar_abs_ backpressure		1.95 ± 0.03
		100% RH, 2.0 bar_abs_ backpressure		2.65 ± 0.04
	+SLG (TT)	100% RH, 1.5 bar_abs_ backpressure		1.58 ± 0.08
		100% RH, 2.0 bar_abs_ backpressure		2.32 ± 0.03

a Errors in the permeability derived from the LSV data are based on the absolute deviation from the mean value of the current density at 0.4 V. Errors in the permeability measurements via MS are based on the standard deviation of the linear fit needed to extract the permeability coefficient. A pressure range of 1–4 bar_abs_ of hydrogen partial pressure was used for the permeation rate measurements (see Figure 2c), whereas compensation for the water vapor pressure was added (see the Experimental Methods).

These values do not comply with the permeation data recorded under dry conditions (Figure 2c) and literature reports about similar SLG-sandwich composite membranes, where, e.g., Bukola et al.^18^ suggest an 8-fold reduction of hydrogen crossover compared to a reference without SLG in a hydrogen pumping cell setup and Chaturvedi et al.^17^ suggest a reduction in hydrogen crossover by around 50% in a custom-made setup. Those permeation measurements were executed in custom-made cells with small active areas (i.e., <0.5 cm^2^) and under close to atmospheric conditions. The cell area can have an influence on the probability of cracks and defects, which increases for bigger areas during the CVD deposition as well as during the transfer process of SLG.^24^ The more dominant effect will most likely stem from the different operating conditions as most of the studies apply a temperature around 25–30 °C. Although the gases were humidified, the absolute water content of the streams at such low temperatures is less than 10% compared to humidified operation at 80 °C. Nevertheless, Kutagulla et al.^19^ reported a reduction in hydrogen crossover of 46%, which was measured at 80 °C and 100% RH. They applied SLG close to the anode, with a 200 nm thin Nafion coating between the anode catalyst layer and the membrane, instead of incorporating SLG between two Nafion membranes. Thus, we hypothesize that the position of the SLG layer in the PEM can influence the integrity of the SLG layer during PEM fuel cell operation, as the mechanical stress on the SLG will change.

Besides the effect of the net humidification state on gas permeability, the mechanical stress caused by swelling and shrinking of the membrane (e.g., during the transfer process or caused by the different thermal expansion coefficient of SLG vs PFSA^16^) as well as by the water drag across the composite membrane during operation has to be considered as it might induce damage to SLG, which further reduces the blocking effect of the monolayer.^6^ It is therefore important to also look at the effect of SLG on water management. Due to the problematic transfer route of the SLG (CT) with respect to electrochemical performance and as the hydrogen crossover reduction is very similar between both SLG composite membranes, the SLG (TT) composite will be discussed further, exclusively.

Defect-free SLG is permeable for protons but blocks all larger atoms and molecules, including water. Therefore, not only is it a barrier for hydrogen gas, but it should also hinder water from traveling through the membrane. The water management within an LT-PEMFC is crucial for its performance.^45−47^ The membrane relies on hydration to enable proton conductivity via vehicular transport and the Grotthuss mechanism.^46^ Further, water diffusion is required to counteract the electroosmotic drag during operation.^47^ A water-impermeable interlayer within a membrane locally blocks the electroosmotic drag, which may reduce proton conductivity by local drying between the interlayer and the cathode. On the other hand, at high loads, the back-diffusion of water could be hindered, which may aggravate the cathode flooding of the fuel cell. We performed hydrogen pumping experiments to evaluate possible changes in the water management of the cells due to the incorporation of SLG. The polarization data of hydrogen pumping mainly show the membrane’s resistance contribution, given the fast kinetics of the hydrogen oxidation and evolution reactions. Hydrogen pumping does not result in net water production like the full fuel cell reaction, but high current densities and a significant electroosmotic drag can be created. Consequently, this experimental approach was chosen as a model to evaluate whether an increase in resistivity occurs in a composite membrane with SLG upon increasing the load under conditions that require ideal water management.

**Figure 7:**
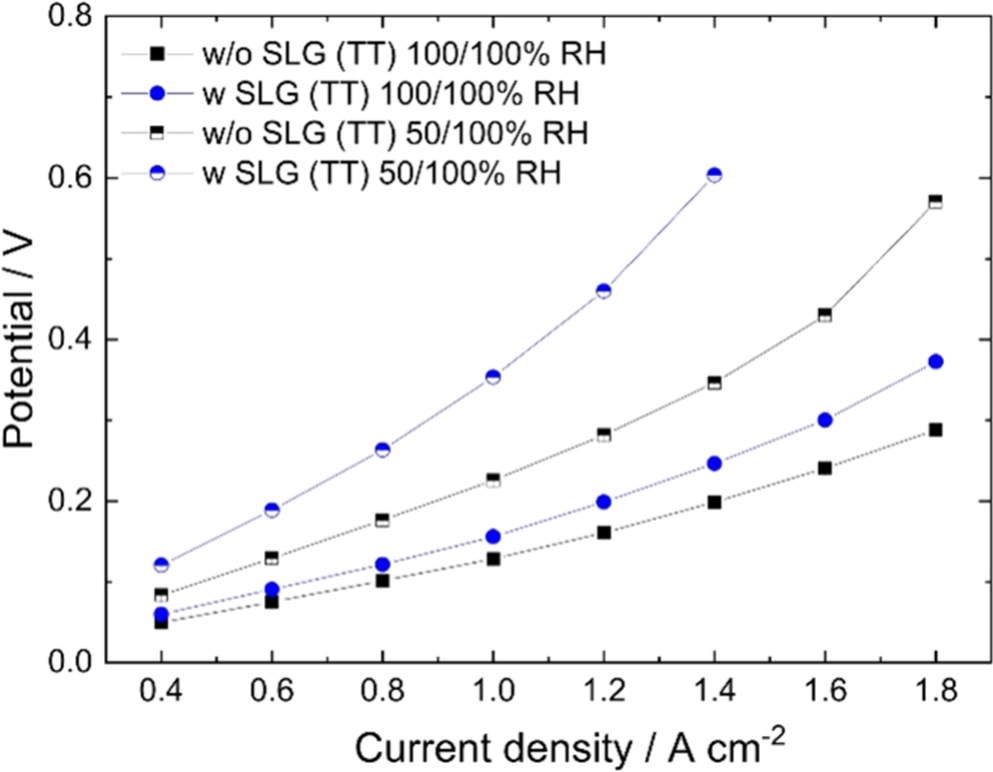
Hydrogen pumping results for an MEA assembled with a double NXL reference membrane without SLG (black) and for an MEA assembled with an SLG (TT) composite membrane (blue), where SLG (TT) is placed in the middle of 2 NXL membranes. H_2_ gas flows were 0.25 l min^–1^ in both anode and cathode compartments. The fuel cell anode (working electrode; HOR) was set to 100% RH, and the cathode (reference electrode; HER) was set to either 100% RH or 50% RH. The experiments were conducted at 80 °C and 1.5 bar_abs_. The MEA with the SLG (TT) composite membrane reached the upper potential limit of 1 V when attempting to run the cell at 1.6 A cm^–2^ for the 50/100% RH experiment. Represented are the average potential values of the current density holds with the standard deviation indicated by error bars.

Hydrogen pumping at full humidification showed a slightly steeper slope of the polarization curve of the MEA assembled with SLG (TT) between 2 NXL membranes compared to the double NXL reference without SLG, with an increasing trend at increasing current densities (Figure 7). Thus, the experiment was repeated with partial humidification at the cathode side, which resulted in the cell performance being more dependent on water drag from the anode to the cathode. In this configuration, the higher resistivity of the SLG (TT) MEA with an increasing trend at increasing current density was more pronounced, resulting in reaching the voltage threshold when attempting to apply more than 1.4 A cm^–2^ (see the Supporting Information for more details; Table S2). These findings indicate that SLG not only acts as a barrier for hydrogen but also for water molecules. Notably, this negative effect on water management is not visible in the fuel cell measurements (Figure 4b), which can be explained by the full humidification of both gas streams and the additional water formation reaction at the cathode.

In the case of defect-free SLG within the MEA, both hydrogen blocking and water blocking are expected to be more pronounced. Therefore, the effect of a water-impermeable interlayer on the water management of the cell is expected to scale accordingly. Thus, these results indicate that the better a hydrogen-blocking interlayer works, the more detrimental side effects on the performance of a humidification-dependent PEM have to be expected.

Finally, the discrepancy between the hydrogen-blocking ability of the SLG interlayer in the hydrogen permeation experiments of the dry membranes (Figure 2c) and in the hydrogen crossover measurements of the operating PEMFC (Figure 6) needs to be evaluated. The two experiments vary in the hydration state of the membranes and the assembly of the membranes with electrodes to form an MEA. Therefore, we executed the hydrogen permeability measurement of the double NXL reference membrane and the SLG (TT) containing double NXL membrane in the as-prepared state as well as of the corresponding MEAs that have undergone fuel cell testing. Measurements were executed at 0% RH, 100% RH, and again 0% RH to check for reversible and irreversible changes in the blocking ability upon membrane hydration. Permeability coefficients are further summarized in Table 2.

**Figure 8:**
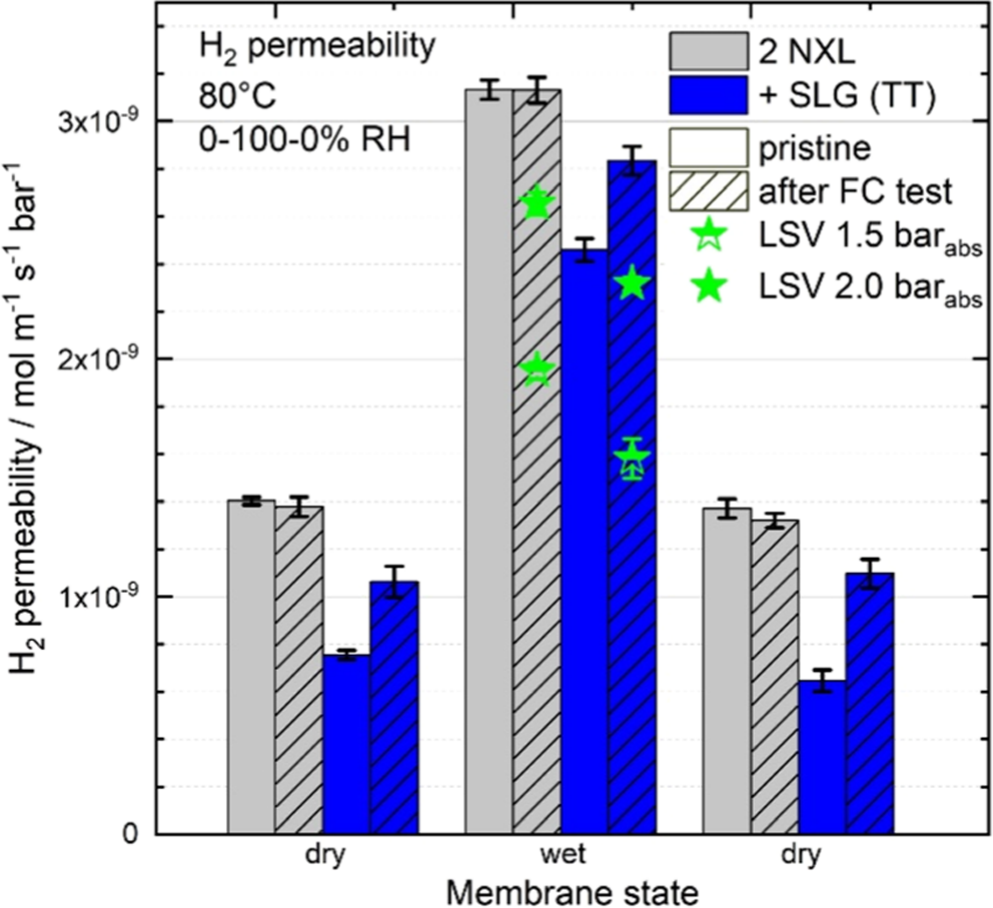
Hydrogen permeability measured via MS of 2 NXL (gray) and 2 NXL + SLG (TT) (blue) for the pristine membranes (unpatterned) and the MEAs that have undergone fuel cell testing (line patterned) at 80 °C either at 0% RH of the gases (“dry”) or 100% RH (“wet”). Permeability coefficients are extracted from a linear fit of the permeation rates over the pressure range of 1–4 bar_abs_ of hydrogen partial pressure (see Figure 2c). Permeabilities estimated by the LSV analysis of the fuel cell measurements (Figure 6) are added as star symbols. All permeabilities are referenced to their dry nominal thickness of 55 μm for both membrane types.

The permeability data (Figure 8) reveals that the hydrogen permeability of fully humidified PFSA membranes is twice as high as the value for dry membranes (gray unpatterned dry and wet), which is in line with the literature.^22^ It can be seen that the hydrogen-blocking effect of SLG reaches about 50% for membranes in the dry status but is limited to only about 20% less than the double NXL reference when the membrane is fully humidified (unpatterned gray vs blue), which confirms the lower reduction that was observed in the fuel cell measurements. The limited H_2_ blocking effect of SLG can be explained by defects and cracks in the SLG and the lateral opening of those cracked layers while humidification of the composite membranes. These defects can be introduced during the large-area CVD^10^ and transfer process,^10,24^ because of the different thermal expansion coefficients^16^ and by differences in the swelling properties of SLG and the NXL membranes.

Dry–wet–dry cycling does not change the double NXL reference membrane characteristics and does not irreversibly damage the hydrogen-blocking ability of SLG in the composite membranes, at least over short time spans, which can be seen when comparing the values obtained in the dry state before and after humidification (i.e., compare first set of columns, left “dry” and third set of column, right “dry” in Figure 8). We assign this to the reversible in-plane swelling of the NXL membranes that allows defect sites of the SLG to reversibly open up laterally and therewith reduce the hydrogen-blocking ability. Notably, the permeabilities of the double NXL reference membrane and the double NXL reference MEA are very similar, which indicates that the MEA fabrication and the fuel cell tests do not significantly impair the gas tightness of a PFSA membrane without SLG (unpatterned gray and patterned gray). On the other hand, the hydrogen permeability of the SLG (TT) MEA is larger than that for the SLG (TT) membrane that did not undergo MEA fabrication and cell testing (patterned blue vs unpatterned blue). Already under dry conditions, the permeability reduction decreases from 49 ± 3% for the pristine SLG composite membrane (unpatterned gray vs blue, dry) to only 20 ± 3% for the corresponding MEA after fuel cell operation (patterned gray vs blue, dry), which is decreasing further under wet conditions to only roughly 10% (patterned gray vs blue, wet). Thus, the permeation results also show that there is an irreversible damage of the SLG that stems from MEA fabrication, fuel cell testing, or both. Figure 8 additionally contains the hydrogen permeabilities of the double NXL reference and SLG (TT) MEAs that were calculated based on the LSV measurements from fuel cell testing (stars). The absolute values of these measurements differ from the results derived from the dedicated permeation setup as they were obtained by a different, indirect method but show similar trends. The SLG (TT) MEA shows about 12–19% reduced hydrogen permeability compared with the double NXL reference without SLG at 80 °C and under full humidification.

Therefore, we conclude that the reduced blocking ability of SLG in composite membranes during fuel cell operation is a combination of reversible losses due to membrane humidification and irreversible defect formation in SLG caused by the stress during MEA fabrication or fuel cell operation.

## Conclusions

In this study, the effect of SLG as a hydrogen-blocking interlayer in PEMs for their application in low-temperature fuel cells was investigated. SLG was incorporated into 5 cm^2^ single cells and operated under standard fuel cell conditions at 80 °C and 100% RH. Two different transfer routes for SLG from either copper or a polymeric substrate were studied. The trivial transfer graphene interlayer in the tested MEAs did not notably affect the performance or proton conductivity of the fuel cell and clearly exceeds the MEAs with an SLG interlayer transferred directly from a copper substrate regarding overall electrochemical performance. The loss in performance of the latter was traced back to a combination of leftover copper ions hindering proton transport in the membrane and remnants of the etching solution poisoning the active platinum sites. An intensive cleaning procedure, for example, boiling the membrane in water or H_2_SO_4_, can help with removing remnants from the etching process. On the other hand, repeated swelling and shrinking will occur when performing these cleaning steps, which could additionally damage the SLG interlayer. Since the transfer of graphene from a polymeric substrate, like trivial transfer graphene, does not result in membrane contaminations in the first place, this route was found to be more viable than the direct transfer from a copper substrate. Hence, it can be concluded that the transfer process has to be thoroughly evaluated and chosen carefully to avoid negative effects on the resulting cell.

A comparably low hydrogen-blocking effect of SLG of only 17 ± 2% at 1.5 bar_abs_ backpressure was achieved in the fuel cells. On the other hand, permeation measurements using MS revealed that the incorporation of SLG in membranes reduces the hydrogen permeation by roughly 50%. This effect, however, is limited to dry membrane conditions, as the membrane swelling by full humidification leads to a reduced blocking ability of only ∼20% compared with the reference. We find that the influence from membrane hydration is reversible but that either MEA fabrication, fuel cell testing, or both result in an additional, irreversible loss in hydrogen-blocking ability. The hydrogen permeability of SLG-containing MEAs after fuel cell testing was reduced by only ∼10% compared with a reference without SLG. Comparing these results with previously published in situ PEMFC results, we believe that not only the membrane hydration but also the position of the SLG layer influences its blocking capability.

Finally, we could observe a water-blocking effect of the SLG interlayer in a particularly water-transport demanding hydrogen pumping experiment, which hints toward the difficulty in finding a gas-blocking layer in solid polymer electrolytes that require water transport in through-plane direction for water management.

Taken together, SLG as a gas-impermeable interlayer in the middle of a PFSA-based membrane showed a significant hydrogen-blocking effect at dry conditions and only a minor effect in realistic cell setups. It is expected to show significant side effects with respect to water management in the case of higher intactness, which would, however, be required for a more substantial hydrogen permeation reduction.

## Supporting Information Available

Raman through-plane scans of reference and SLG composite membranes; photographs of the two SLG transfer methods onto Nafion XL; linear sweep voltammetry (LSV) scan before the fuel cells break in Raman analysis of SLG (TT) on different substrates and conditions; in-plane Raman analysis of SLG (TT) on quartz and Nafion XL; cross-sections of all MEA types after fuel cell tests and membrane thickness evaluation; calculation of the excess ratio of the etchant during the SLG transfer from copper; ICP-MS analysis and the calculation of the minimum copper contamination of the membrane after transferring SLG from the copper substrate; calculation of the permeability coefficients from LSV measurements; table of membrane resistivity calculated from the hydrogen pumping experiments (PDF)
