# Supplementary material for: Applicability of Single-Layer Graphene as a Hydrogen-Blocking Interlayer in Low-Temperature PEMFCs
Source: ACS Appl Mater Interfaces. 2024 Apr 27;16(18):23220–32. doi: 10.1021/acsami.4c01254 (PMC11082842; doi:10.1021/acsami.4c01254)
Supplement: Supplementary file 1 — am4c01254_si_001.pdf [file am4c01254_si_001.pdf]

Supporting Information

Applicability of Single-Layer Graphene as a Hydrogen-Blocking Interlayer in Low-Temperature PEMFCs

Miriam Komma[1,2], Anna T.S. Freiberg[1,2], Dunia Abbas[1,2], Funda Arslan[1,2], Maja Milosevic[1,2], Serhiy Cherevko[1], Simon Thiele[1,2] and Thomas Böhm[1,]*

[1] Forschungszentrum Jülich GmbH, Helmholtz Institute Erlangen-Nürnberg for Renewable Energy (IEK-11), Cauerstr.1, 91058 Erlangen, Germany

[2] Department of Chemical and Biological Engineering, Friedrich-Alexander-Universität Erlangen-Nürnberg, Cauerstr.1, 91058 Erlangen, Germany

*corresponding author e-mail: t.boehm@fz-juelich.de

S 1 Additional information on Raman through-plane scans and on the manufacturing of Single-Layer Graphene (SLG) Nafion^{TM} composite membranes

S 1.1 Raman through-plane scans of reference and SLG composite membranes

Raman analysis was performed to confirm the successful transfer of SLG onto Nafion^{TM} XL (NXL) membranes. Figure S1 shows the unprocessed sum filter images of the hyperspectral Raman through-plane scans of a NXL membrane before and after hotpressing and of the composite membranes NXL + SLG (TT) and NXL + SLG (CT).

Notably, the Raman image of SLG (CT) on NXL shows a reduced signal-to-noise ratio compared to SLG (TT) on NXL. This phenomenon results from a pronounced background in the spectra of the membrane sample with SLG (CT). This effect did not occur in hotpressed NXL without a SLG transfer (see Figure S1) and can therefore be explained by remnants of copper or the etchant solution in the SLG (CT) membrane sample.

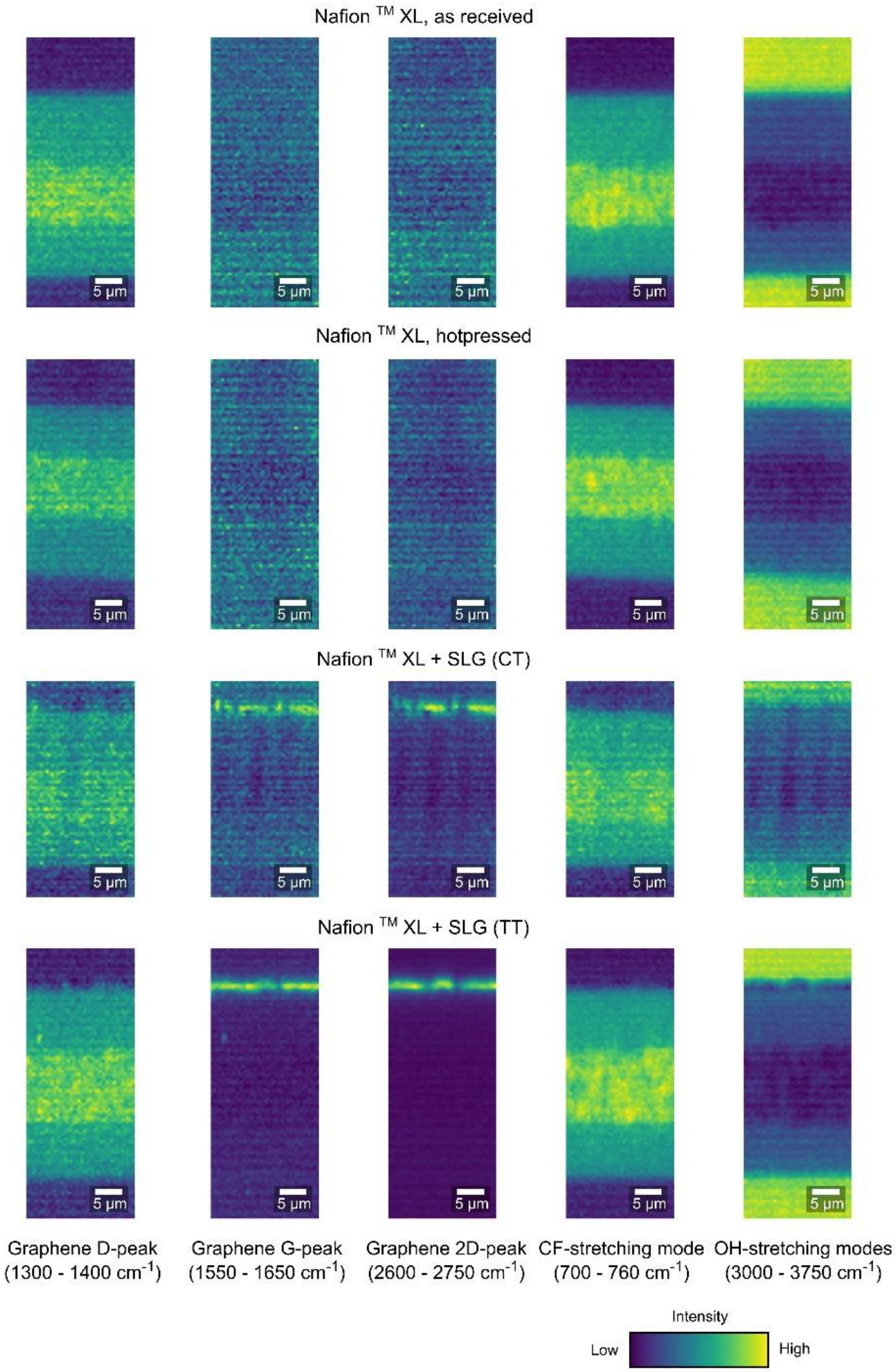

Figure S1. Unprocessed sum filter images of the hyperspectral Raman through-plane scans of Nafion™ XL (NXL) membranes before and after hotpressing and with SLG transferred onto NXL from trivial transfer™ graphene (SLG (TT)) and from a copper substrate (SLG (CT)). The spatial intensity distribution is shown for graphene-related bands (D, G, and 2D) as well as typical signals of the CF- and OH- stretching modes of PFSA/PTFE and water. The D-peak sum filter image primarily shows signals from the ionomer due to the overlap with Raman bands of Nafion^{TM}. Weak D-band related signals are sparsely visible at the locations of SLG (CT) and SLG (TT).

S 1.2 Photographs of the two SLG transfer methods onto Nafion^{TM} XL

Figure S2 shows photographs taken at different steps during the graphene transfer processes. The upper row shows the SLG (CT) method, and the lower row depicts the SLG (TT) approach.

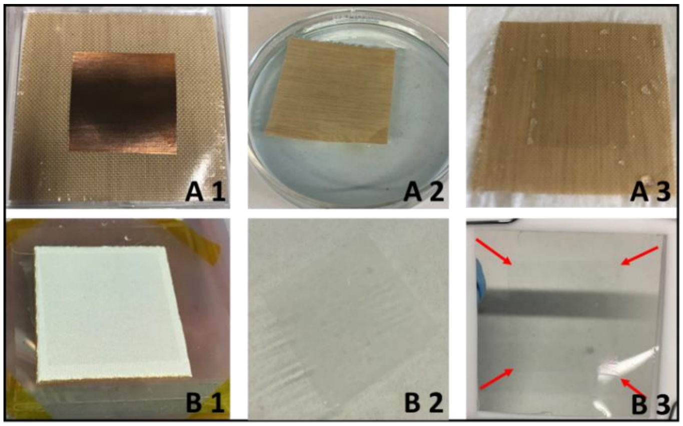

Figure S2. Photographs of the two different transfer methods of single-layer graphene (SLG) onto PFSA membranes. The transfer method using SLG deposited on a copper substrate is shown in the upper panel. A1: hotpressing of SLG onto Nafion™ XL, A2: the etching process in ammonium persulfate (APS), and A3: rinsing of the Nafion™ XL+ SLG membrane with DI water. The lower panel shows the transfer process of trivial transfer™ graphene from ACS Material LLC. B1: trivial transfer™ graphene as received, B2: trivial transfer™ graphene floating on DI water, and B3: trivial transfer™ graphene on Nafion^{TM} XL after removing of the polymethylmethacrylate (PMMA) layer with ethyl acetate and drying of the Nafion™ XL+ SLG membrane.

S 2 Linear sweep voltammetry (LSV) scan before the fuel cells break in

Hydrogen crossover analysis was done prior to the break-in procedure and performance test of the fuel cell to exclude the impact of those processes on the SLG interlayer. We investigated the hydrogen crossover with LSV scans under fully humidified conditions at 80°C and 1.5 bar_{abs}, which are shown in Figure S3. Since the MEA did not undergo any break-in protocol prior to this analysis, the increasing current with increasing potential can be explained by the pristine state of the MEAs. Nevertheless, the reduction in hydrogen crossover of 12-17% (calculated by the mean crossover current densities at 0.4 V from the LSV measurements) by the SLG remains similar to the reduction after the break in and performance analysis (15-19%) of the MEAs shown in the main text.

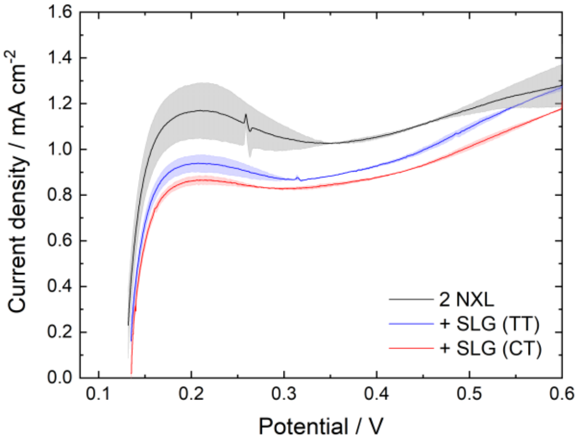

Figure S3. Evaluation of the LSV measurements of the reference (2 NXL) MEAs and the SLG (TT) and SLG (CT) composite MEAs before the break-in procedure and performance tests of the MEAs. LSVs were conducted after 1 h constant flow of 1 l min^{-1} H_2 (100% RH) and N_2 (100% RH) to ensure decent protonic conductivity and humidification. LSVs were performed at flow rates of 0.2 l min^{-1} H_2 at the fuel cell anode compartment and 0.2 l min^{-1} N_2 at the fuel cell cathode compartment. All experiments were performed at 80 °C, 1.5 bar$_{abs}$ 100% RH. Represented are the mean values with the absolute deviation indicated by the shaded area of two independently measured MEAs per sample type.

S 3 Additional Raman analysis of SLG (TT)

S 3.1 Raman analysis of SLG (TT) on different substrates and conditions

The ratio between G- and 2D-band is not equal for the SLG (CT) and SLG (TT) composite membranes (Figure 2). However, the ratio between these two peaks is sensitive to the surrounding of SLG [S1], and we found changes in this ratio depending on the presence of PMMA, Nafion™, and water (Figure S4). Defects in the SLG structure and remnants like copper ions from the etching process in the SLG (CT) transfer might also influence the ratio of those two bands.

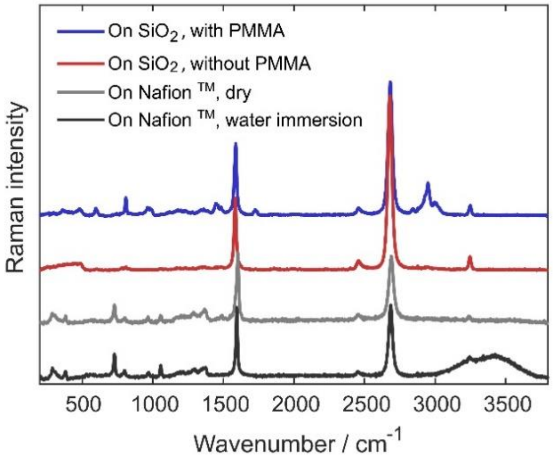

Figure S4. Raman spectra of SLG on different substrates and in different conditions. Raman spectra of SLG (TT) on SiO_2 substrate (quartz) with PMMA coating, SLG (TT) on SiO_2 after removing the PMMA coating, a dry SLG (TT)-Nafion™-composite membrane, and an SLG (TT)-Nafion™ composite membrane immersed in water, were acquired.

The spectra are normalized to the maximum intensity of the G peak between 1550 and 1650 cm^{-1} to analyze the impact of different environments regarding substrate, coating and dry or water immersed on the spectral bands of SLG. The spectra show varying ratios between 2D (around 2685 cm^{-1}) and G (around 1590 cm^{-1}) peaks of SLG. In addition, Raman bands of the surrounding molecules (PMMA, Nafion, and water) are visible due to the diffraction-limited resolution of confocal Raman microscopy.

S 3.2 In-plane Raman analysis of SLG (TT) on quarz glas and on Nafion^{TM} XL

The following figures (Figure S5 – S10) show in-plane Raman images of SLG (TT) on quartz (Figure S5, S6, S7) and on Nafion^{TM} XL (Figure S8, S9, S10), each taken at a different location, to assess the quality of the SLG (TT) after transfer. The scans were taken as stacks along the through-plane direction to account for tilted samples since the signal-to-noise ratio of SLG depends on the focus position of the microscope (SLG is already out-of-focus when the focus plane is approx. 1 µm below or above the SLG). The scans on quartz slides were performed with a high NA dry objective (100x/0.9) since this objective provides a very high resolution, and the scans on Nafion^{TM} XL were performed with a water immersion objective that enables a superior signal-to-noise ratio and helps with preventing thermal degradation of the samples. The scans were obtained with the WITec alpha 300 confocal Raman microscope using a 532 nm laser and a UHTS VIS spectrometer (600 grooves/mm optical grating and a Peltier-cooled EMCCD camera). The laser power was adjusted for both sample types to prevent thermal degradation: The SLG (TT) on quartz was imaged at 25 mW laser power and the SLG (TT) on Nafion^{TM} XL at 10 mW. The pixel size was 500 nm and the integration time was 50 ms per pixel, and the sum filter images were prepared after background subtraction and cosmic ray removal.

The scans reveal that SLG (TT) is homogeneously present over the whole imaged area (25x25 μm^2 per mapping) according to the G and 2D peak sum filter images. However, it can be noted that there are local intensity deviations within these sum filter images, which correlate with the presence of D peak bands. This phenomenon is clearly visible in the images of SLG (TT) on quartz (Figure S5, S6, S7). Locally present D peak bands cannot be resolved in the images of SLG (TT) on Nafion^{TM} XL (Figure S8, S9, S10), which can be traced back to the spectral convolution of the D peak of SLG and the fingerprint spectrum of Nafion^{TM}. The Raman images show that SLG was successfully transferred onto Nafion^{TM}, but they cannot display the presence of cracks within or small gaps between SLG flakes below the lateral resolution limit of confocal microscopy (< around 500 nm).

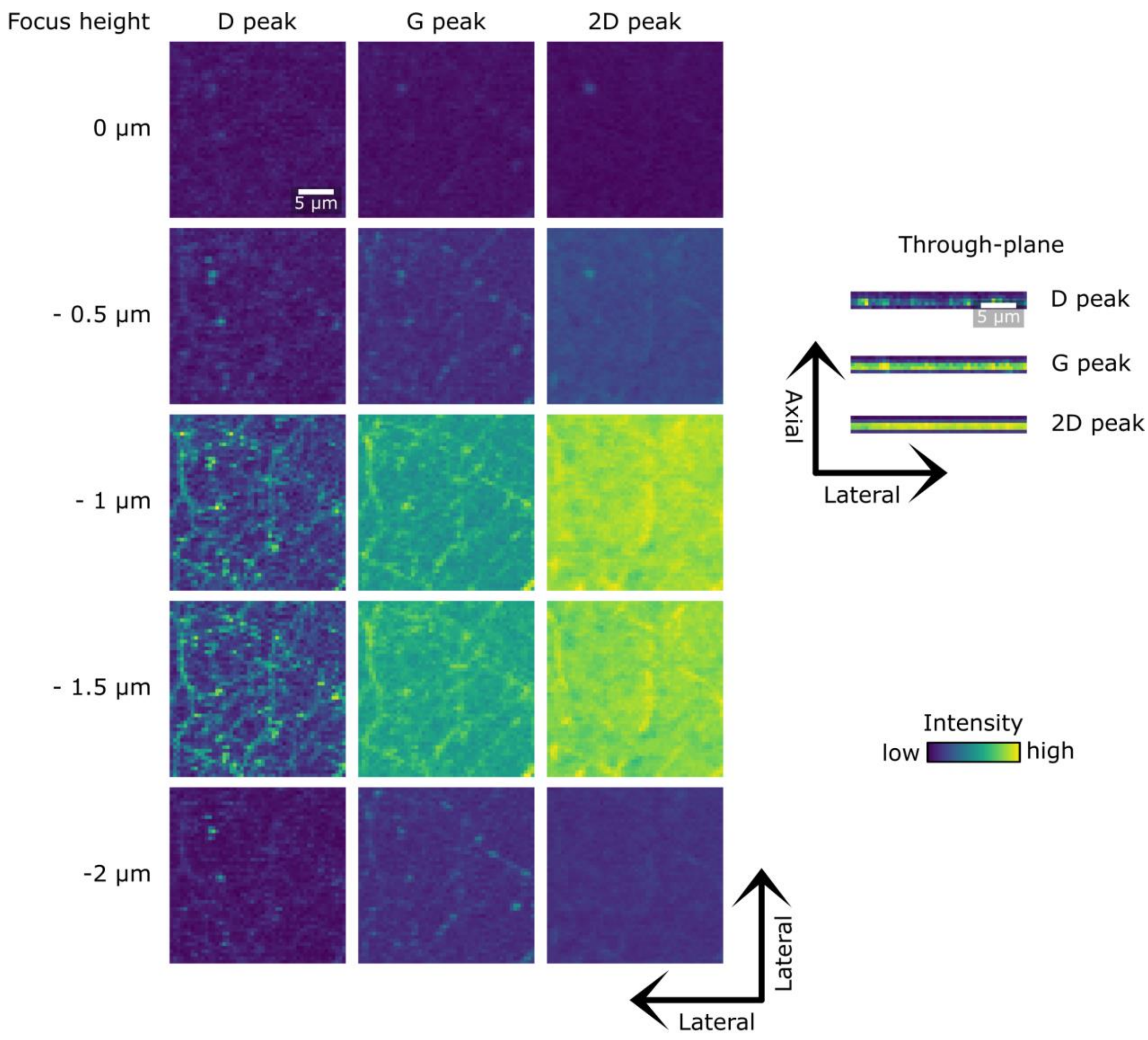

Figure S5. Raman images of SLG (TT) on quartz (spot 1). Hyperspectral Raman images were obtained using a 532 nm laser and a 100x/0.9 objective (dry) at a pixel size of 0.5 µm. The images show sum filters for the D peak (1300-1400 cm^{-1}), the G peak (1550-1650 cm^{-1}), and the 2D peak (2600-2750 cm^{-1}) after background subtraction. The intensity of each sum filter panel is normalized to the maximum intensity and minimum intensity within this stack. The images on the left depict in-plane scans, and the images on the right are a single through-plane cross-section of the 3D image stack.

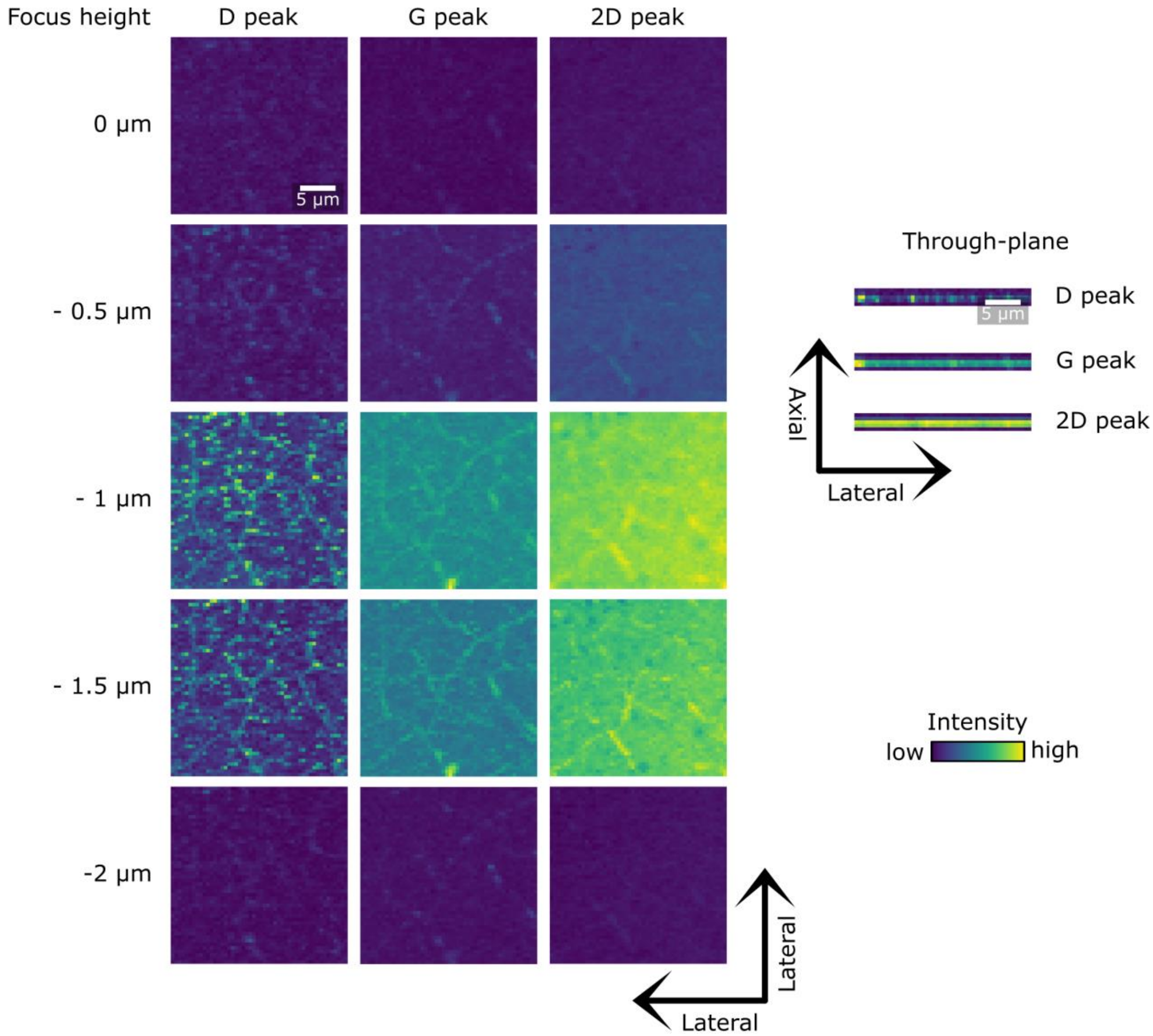

Figure S6. Raman images of SLG (TT) on quartz (spot 2). Hyperspectral Raman images were obtained using a 532 nm laser and a 100x/0.9 objective (dry) at a pixel size of 0.5 µm. The images show sum filters for the D peak (1300-1400 cm^{-1}), the G peak (1550-1650 cm^{-1}), and the 2D peak (2600-2750 cm^{-1}) after background subtraction. The intensity of each sum filter panel is normalized to the maximum intensity and minimum intensity within this stack. The images on the left depict in-plane scans, and the images on the right are a single through-plane cross-section of the 3D image stack.

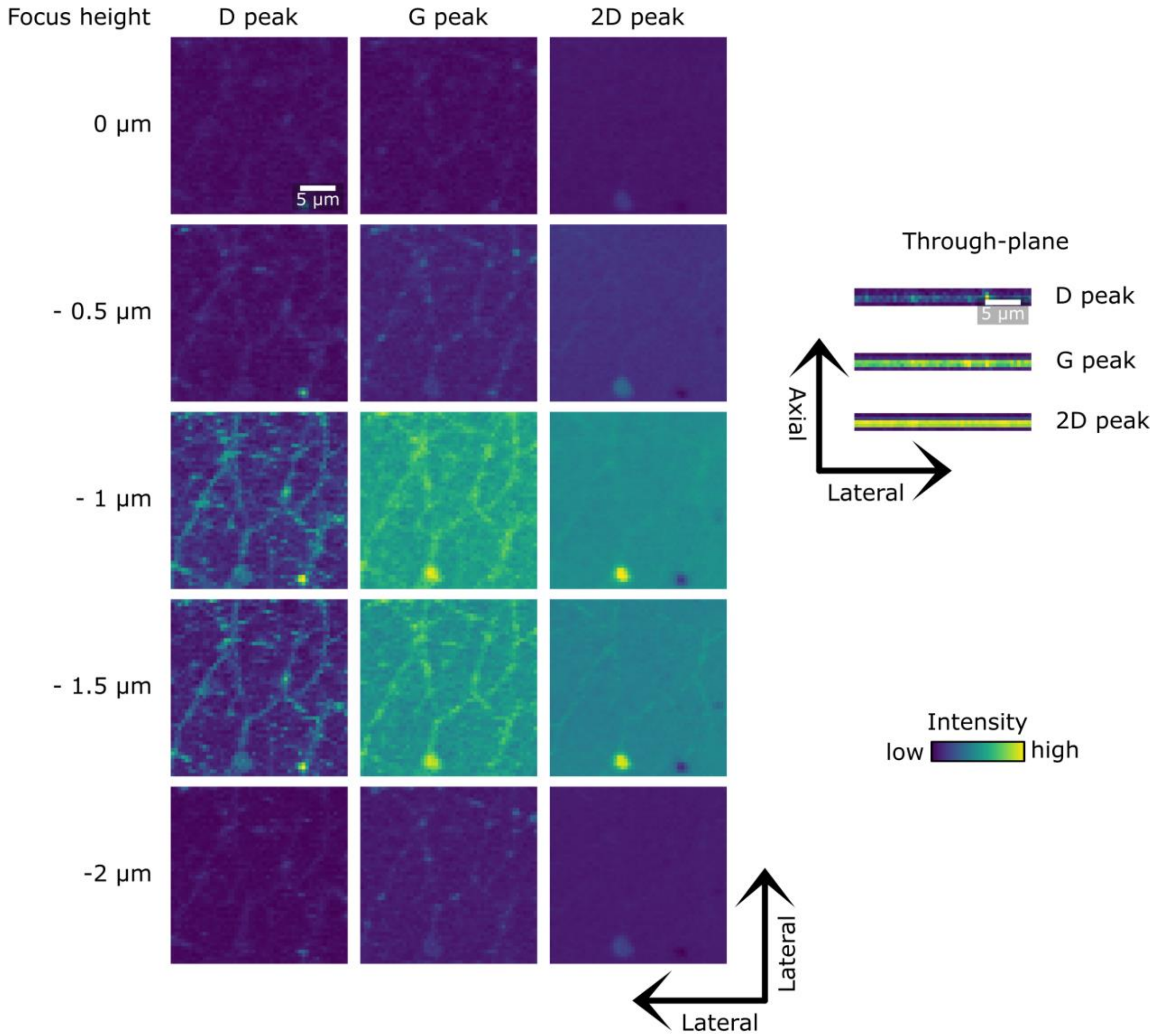

Figure S7. Raman images of SLG (TT) on quartz (spot 3). Hyperspectral Raman images were obtained using a 532 nm laser and a 100x/0.9 objective (dry) at a pixel size of 0.5 µm. The images show sum filters for the D peak (1300-1400 cm^{-1}), the G peak (1550-1650 cm^{-1}), and the 2D peak (2600-2750 cm^{-1}) after background subtraction. The intensity of each sum filter panel is normalized to the maximum intensity and minimum intensity within this stack. The images on the left depict in-plane scans, and the images on the right are a single through-plane cross-section of the 3D image stack.

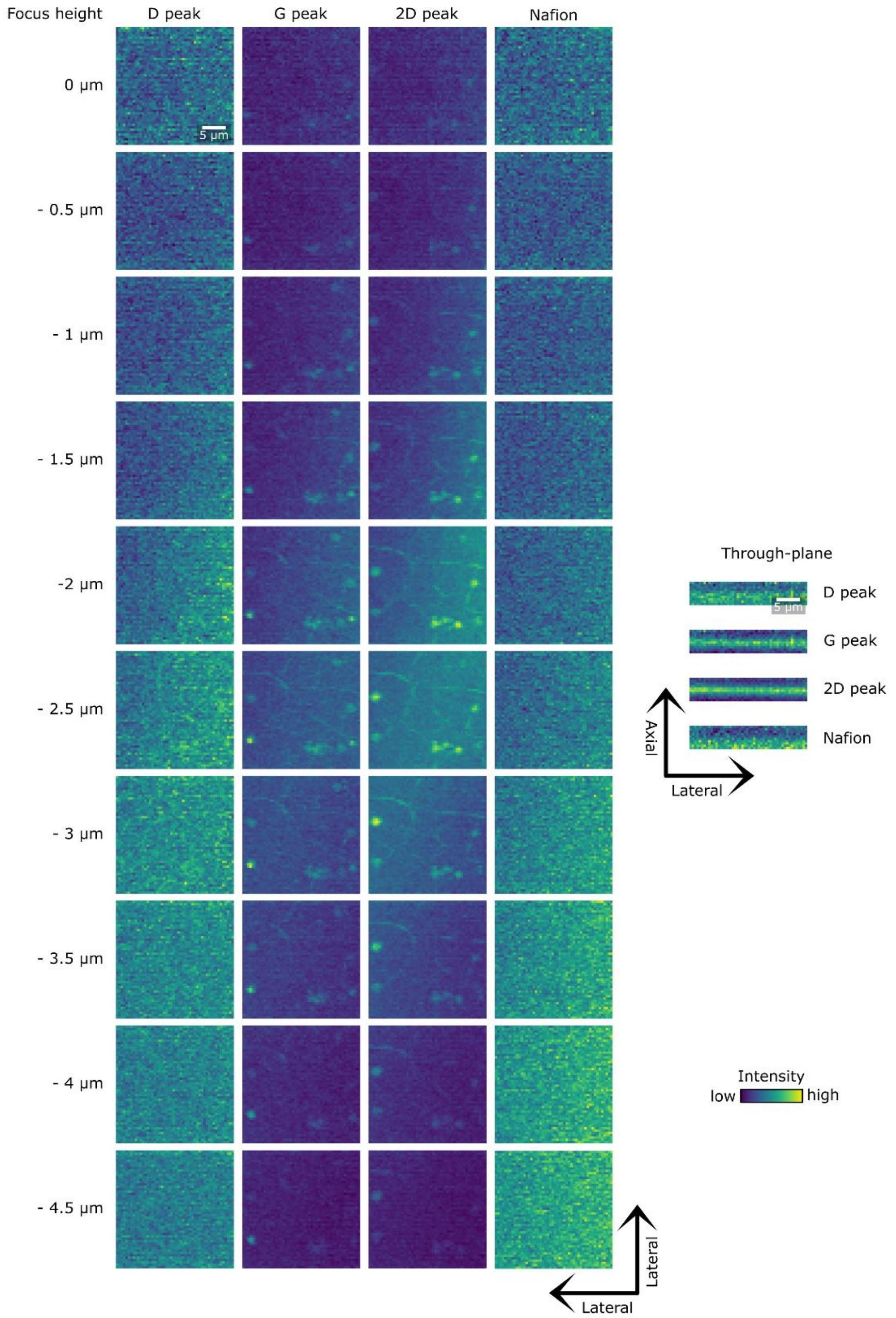

Figure S8. Raman images of SLG (TT) on Nafion™ XL (spot 1). Hyperspectral Raman images were obtained using a 532 nm laser and a 63x/1 objective (water immersion) at a pixel size of 0.5 µm. The images show sum filters for the D peak (1300-1400 cm^{-1}), the G peak (1550-1650 cm^{-1}), the 2D peak (2600-2750 cm^{-1}), and the CF peak of Nafion (700-760 cm^{-1}) after background subtraction. The intensity of each sum filter panel is normalized to the maximum intensity and minimum intensity within this stack. The images on the left depict in-plane scans, and the images on the right are a single through-plane cross-section of the 3D image stack.

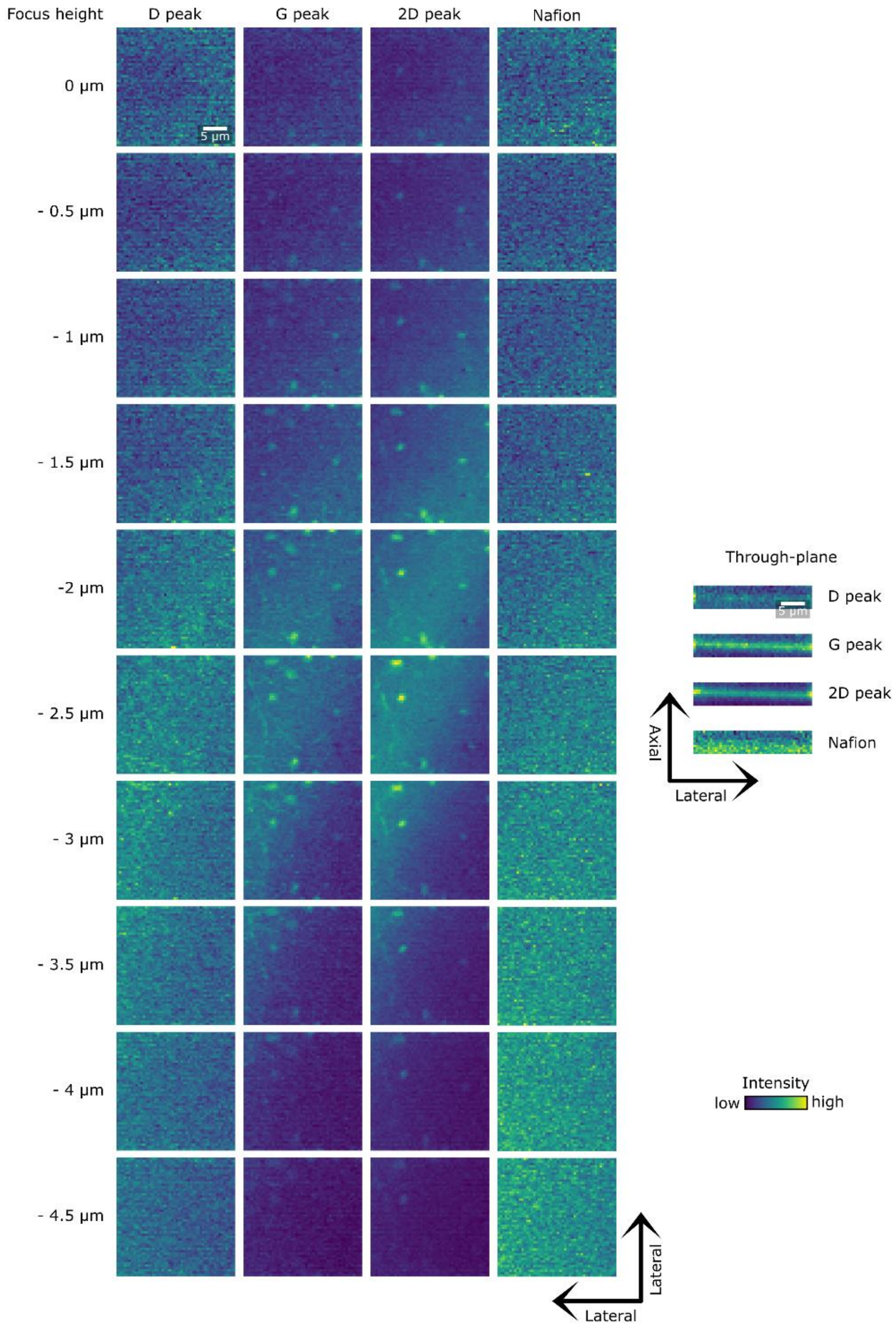

Figure S9. Raman images of SLG (TT) on Nafion™ XL (spot 2). Hyperspectral Raman images were obtained using a 532 nm laser and a 63x/1 objective (water immersion) at a pixel size of 0.5 µm. The images show sum filters for the D peak (1300-1400 cm^{-1}), the G peak (1550-1650 cm^{-1}), the 2D peak (2600-2750 cm^{-1}), and the CF peak of Nafion (700-760 cm^{-1}) after background subtraction. The intensity of each sum filter panel is normalized to the maximum intensity and minimum intensity within this stack. The images on the left depict in-plane scans, and the images on the right are a single through-plane cross-section of the 3D image stack.

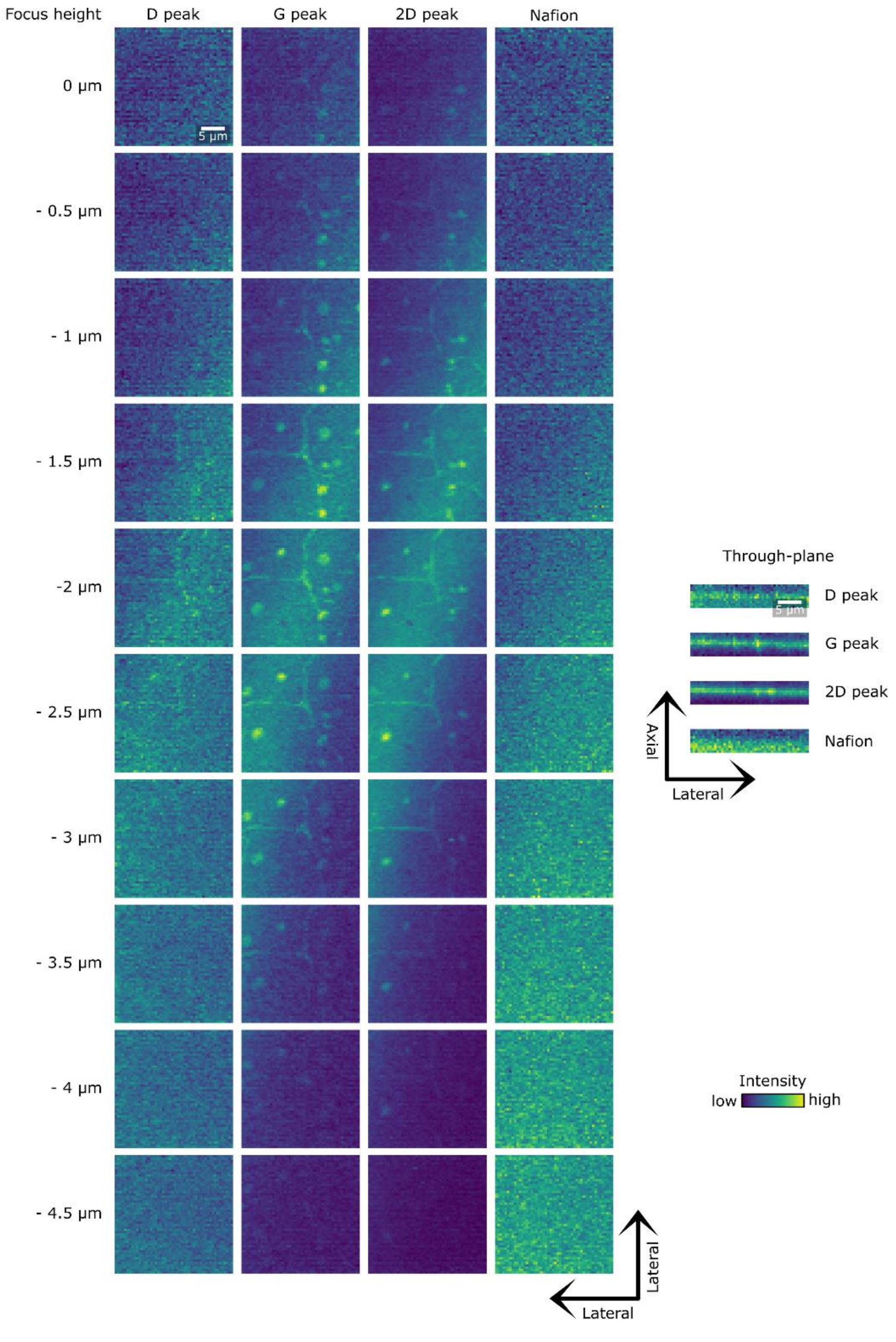

Figure S10. Raman images of SLG (TT) on Nafion™ XL (spot 3). Hyperspectral Raman images were obtained using a 532 nm laser and a 63x/1 objective (water immersion) at a pixel size of 0.5 µm. The images show sum filters for the D peak (1300-1400 cm^{-1}), the G peak (1550-1650 cm^{-1}), the 2D peak (2600-2750 cm^{-1}), and the CF peak of Nafion (700-760 cm^{-1}) after background subtraction. The intensity of each sum filter panel is normalized to the maximum intensity and minimum intensity within this stack. The images on the left depict in-plane scans, and the images on the right are a single through-plane cross-section of the 3D image stack.

S 4 Cross-sections of all MEA types and membrane thickness analysis of the SLG (TT) MEA after hydrogen pumping and of double NXL reference, SLG (TT) and SLG (CT) membranes after fuel cell testing

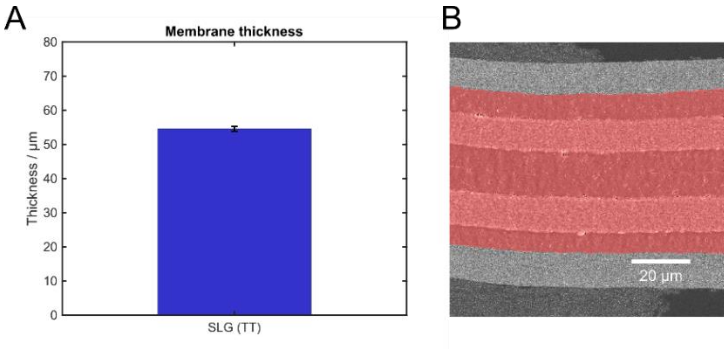

Figure S11. Membrane thickness (A) from the cross-sectional SEM image of the SLG (TT) MEA after hydrogen pumping (B) (compare Figure 3). The thickness of the SLG (TT) MEA after hydrogen pumping was determined to 54.5 µm ± 0.69 µm. The highlighted red area in B is the segmented area of the membrane that was used to calculate the membrane thickness. The data in A is the mean thickness over the full image width, and the error bars denote the standard deviation of the data.

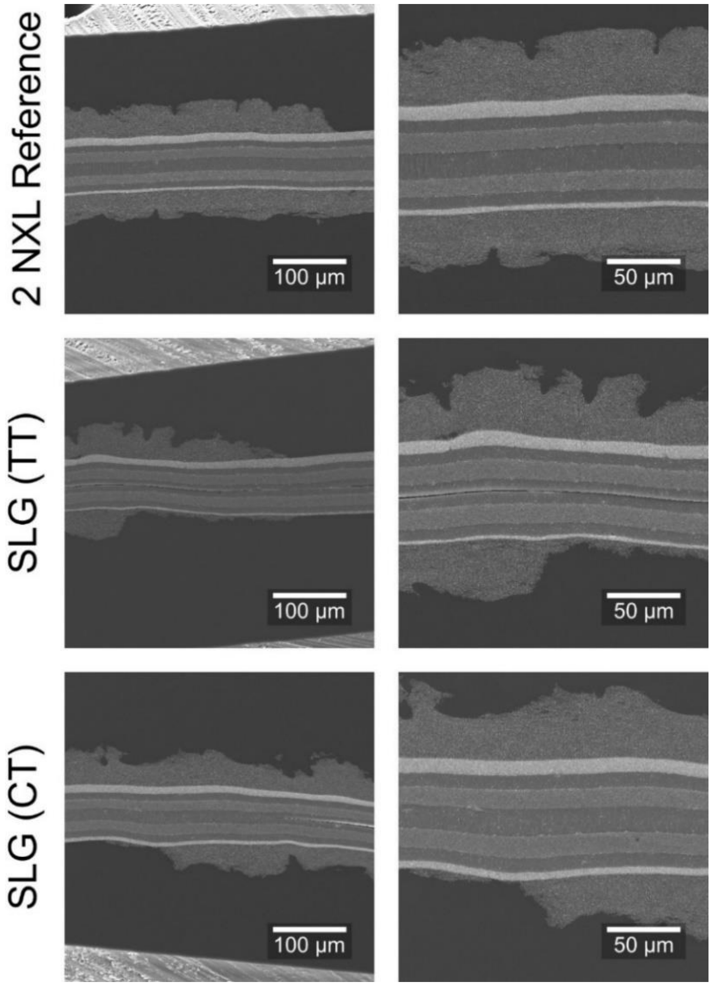

Figure S12. Cross-sectional SEM images of all three MEA types (2 NXL reference, SLG (TT) and SLG (CT)) after fuel cell testing.

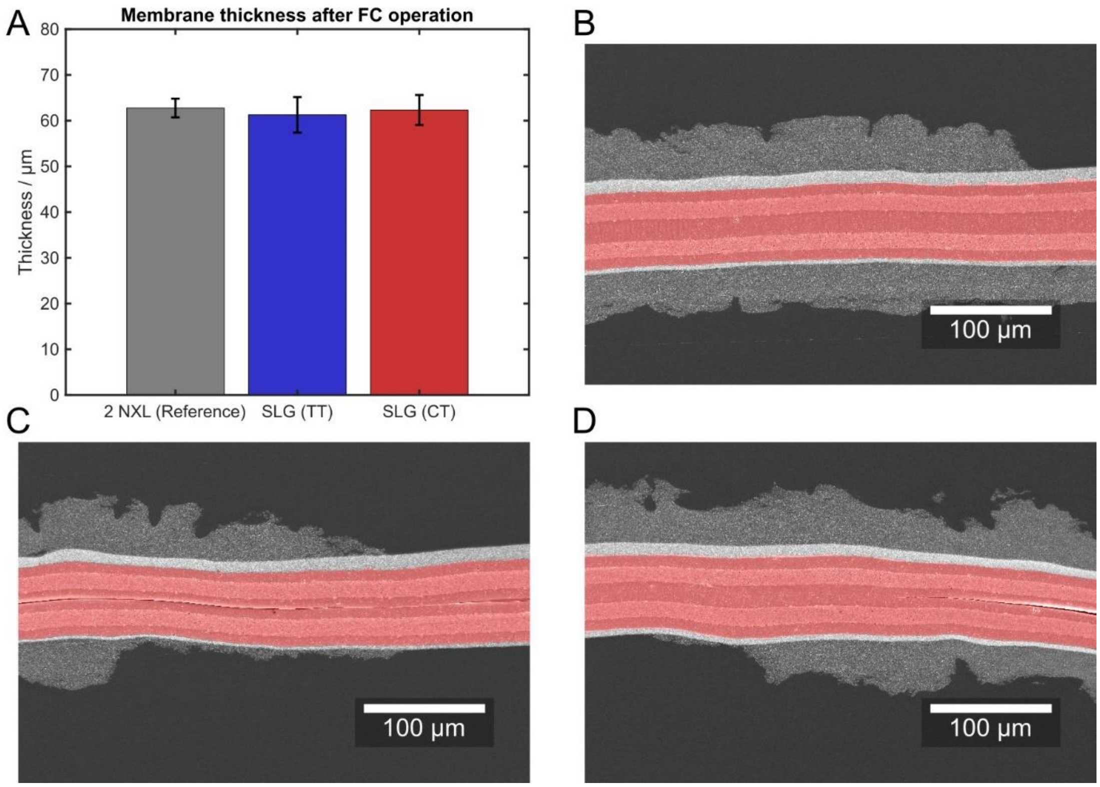

Figure S13. Membrane thickness analysis (A) of all three tested membranes from the cross-sectional SEM images of the MEAs (B: 2 NXL reference; C: SLG (TT); D: SLG (CT)) after fuel cell testing. The highlighted red area in B, C, and D is the segmented area of the membrane that was used to calculate the membrane thickness. The data in A is the mean thickness over the full image width, and the error bars denote the standard deviation of the data.

The membrane thickness evaluation in Figure S11 and Figure S13 were performed by segmentation of the membrane in the SEM images (red areas in the figures) and measuring the thickness over the full image width using Matlab.

S 5 Additional information on the SLG (CT) transfer process from a copper substrate

S 5.1 Calculation of the excess ratio of the etchant during the SLG transfer from copper

The molar amount of copper to be etched can be calculated using the density of copper ρ_{Cu}, the volume of the copper substrate layer V_{Cu} and the molar mass of copper M_{Cu} following Equation S1.

$$n_{Cu}=\frac{\rho_{Cu}\cdot V_{Cu}}{M_{Cu}}=\frac{8.96\frac{g}{cm^3}\cdot(2.54\ cm)^2\cdot 18\cdot 10^{-4} cm}{63.55\frac{g}{mol}}=1.6\ mmol \qquad \text{Equation (S1)}$$

The molar amount of APS used in the etching process can be calculated using the used volume V_{APS} and the molarity of the solution c_{APS} leading to a molar ratio of APS to copper as given in Equation S2.

$$\frac{n_{APS}}{n_{Cu}} = \frac{V_{APS} \cdot c_{APS}}{n_{Cu}} = \frac{50\ \mathrm{ml} \cdot 0.26 \frac{\mathrm{mol}}{\mathrm{l}}}{1.6\ \mathrm{mmol}} = 8 \qquad \text{Equation (S2)}$$

Thus, an 8 times molar excess of APS compared to copper was used during etching of the copper substrate.

S 5.2 ICP-MS analysis and the calculation of the minimum copper contamination of the membrane after transferring SLG from the copper substrate

A calibration curve was measured for the ICP-MS analysis using freshly prepared calibration solutions containing Cu (0.5 to 5 µg l^{-1}) and a blank solution without Cu. Additionally, a Co solution (10 µg l^{-1}) was used as an internal standard. For all calibration solutions, the Cu counts (black squares in Figure S14) were evaluated and defined the calibration curve for the ICP-MS analysis. For detecting remnants of copper in the SLG (CT) composite membrane, a 1 cm^2 sample of the membrane was prepared. For volumetric reasons, the copper substrate was etched with 25 ml of the 0.26 M APS etching solution, keeping the molarity of the etching solution constant but with an increased absolute molar excess of APS. Further, a reference composite membrane was treated equally but without transferring SLG (CT). The samples were soaked in 10 ml of a 0.1 M HCl solution for 24 h at 60 °C to wash out remaining copper ions in the membrane. This 0.1 M HCl washing solution was then analyzed with ICP-MS. For the washing solution of the reference sample, the counts were below the value of the calibration solution without any copper content. Hence it can be considered as 0 µg l^{-1}. For the washing solution of the SLG (CT)-composite membrane, the counts correspond to a copper ion concentration of 1.4 µg l^{-1} (red square S14). However, no full ion exchange can be verified after washing the membrane as described. Therefore, this value is not suitable to quantify the copper contamination of the membrane after transferring SLG (CT) from the copper substrate. Nonetheless, we can provide a minimum value of sulfonic acid groups within the composite membrane being contaminated by copper after transferring SLG (CT) onto the membrane based on the ICP-MS results.

The equivalent weight of NXL was experimentally investigated with Raman spectroscopy to be dependent on the position within the membrane. [S2] In reinforced areas of the NXL an EW of 2000 g mol^{-1} was detected, whereas the not reinforced parts of NXL showed an EW of around 970 g mol^{-1}. As we do not want to hypothesize on the location and mobility of the copper ions within the membrane, we provide an estimate for model membranes with homogeneous EWs of 2000 and 970 g mol^{-1}.

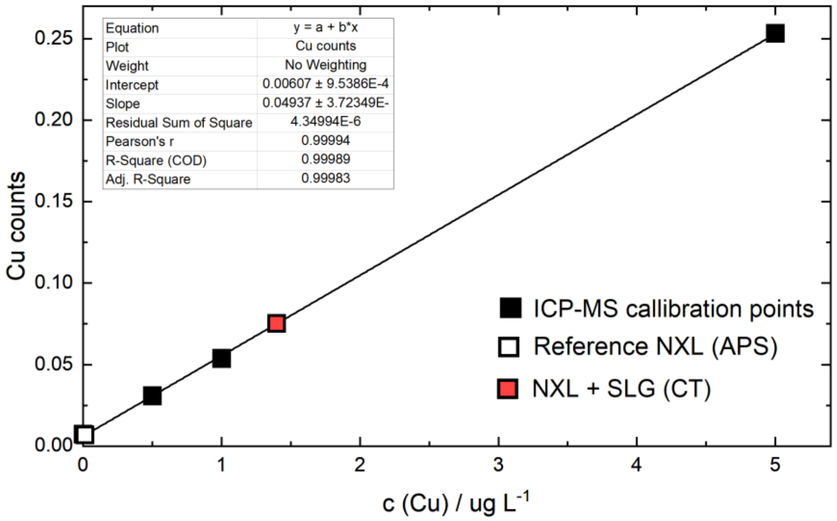

Figure S14. ICP-MS measurements of the reference sample NXL (APS) soaked in APS for 1h and the NXL+ SLG (CT) sample representing the SLG (CT) transfer route. Copper was clearly detected in the NXL+ SLG (CT) sample, while the value of the reference sample was lower than the calibration solution that was not containing any copper ions.

The mass of a 1 cm² piece of NXL $m_{NXL}^{1cm^2}$ membrane can be calculated using the basic weight of NXL (BW_{NXL}) of 55 g m^{-2} [S3] following Equation S3. The minimum and maximum ion exchange capacities $IEC^{min/max}$ are given by the different EWs of the reinforced and not reinforced parts following Equation S4.

$$m_{NXL}^{1cm^2}=A_{NXL}\cdot BW_{NXL}=1\ cm^2\cdot 55\,\frac{g}{m^2}=55\cdot 10^{-4}g \qquad \text{Equation (S3)}$$

$$IEC^{min/max}=\frac{1}{EW^{max/min}} \qquad \text{Equation (S4)}$$

$$IEC^{min}=0.5\ \cdot 10^{-3}\,\frac{mol}{g}$$

$$IEC^{max}=1.0\ \cdot 10^{-3}\,\frac{mol}{g}$$

The minimum and maximum amount of sulfonic acid groups $n_{SO_3^+}^{min/max}$ for the model membranes consisting entirely of EWs of 2000 and 970 g mol^{-1} is estimated using Equation S5.

$$n_{SO_3^+}^{min/max}=IEC^{min/max}\cdot m_{NXL}^{1cm^2} \qquad \text{Equation (S5)}$$

$$n_{min}^{SO_3^+}=2.8\cdot 10^{-6}mol$$

$$n_{max}^{SO_3^+}=5.5\cdot10^{-6}mol$$

Equation S6 provides the amount of copper detected in the SLG (CT) membrane that was analyzed using ICP-MS by evaluating the detected copper concentration.

$$n_{Cu}=\frac{m_{Cu}}{M_{Cu}}=\frac{c_{Cu}^{wash}\cdot V_{Cu}^{wash}}{M_{Cu}}=\frac{1.4\frac{\mu g}{l}\cdot 10\ ml}{63.5\frac{g}{mol}}=2.2\cdot10^{-10}mol \qquad \text{Equation (S6)}$$

Consequently, at least 0.4 in 10^4 (EW970; one Cu ion blocks one sulfonic acid group) up to 1.6 in 10^4 (EW2000; one Cu ion blocks two sulfonic acid groups) sulfonic acid groups are blocked by copper in the SLG (CT) membrane sample. This is a lower limit approximation that likely underestimates the actual value and impact on fuel cell performance due to (i) a possibly incomplete exchange of copper ions during washing, (ii) the possibility of multi-site blocking by multi-valent copper ions, and (iii) the local confinement of copper ions to the outer-most membrane region.

S 6 Calculation of the permeability coefficients from LSV measurements

Permeability coefficients were calculated from the mean crossover current density at 0.4 V from the LSV measurements (after the break-in of the fuel cell) at 1.5 and 2 bar_{abs} according to Equation S7 with the Faraday constant (96485 s A mol^{-1}), the number of transferred charges of 2 and the nominal thickness of 2 NXL membranes of 55 µm. The partial pressure of water at 80 °C of +0.474 bar was taken into account.

$$\varepsilon_{H_2}=\frac{I}{z\cdot F}\cdot\frac{d}{p}=\frac{I}{2\cdot 96485\ s\cdot A\cdot mol^{-1}}\cdot\frac{55\cdot10^{-6}\ m}{(p-0.474\ bar)} \qquad \text{Equation (S7)}$$

Table S1: Permeability coefficients of the reference membrane (2 NXL) and the SLG (TT) and SLG (CT) composite membranes. The permeability coefficients were calculated from the mean crossover current densities at 0.4 V from LSV measurements at 1.5 and 2 bar_{abs} according to Equation S7.

	2 NXL	2 NXL + SLG (TT)	2NXL + SLG (CT)
Pressure	ε_{H_2} / $\frac{10^{-9}\ mol}{m\ s\ bar}$	ε_{H_2} / $\frac{10^{-9}\ mol}{m\ s\ bar}$	ε_{H_2} / $\frac{10^{-9}\ mol}{m\ s\ bar}$
1.5 bar_{abs}	1.95 ± 0.03	1.58 ± 0.08	1.65 ± 0.08
2 bar_{abs}	2.65 ± 0.04	2.32 ± 0.03	2.42 ± 0.01

S 7 Hydrogen pumping experiments

High current densities and significant electro-osmotic drag can be created in hydrogen pumping experiments. These experiments were performed to investigate the impact of SLG on water transport and therefore proton transport within the membrane since SLG theoretically shows high blocking capability even for water molecules. The anode and the cathode compartment of the fuel cell setup were continuously flushed with 0.25 l min^{-1} H_2, the active area was 5 cm^2 and Pt loadings

were 0.3 ± 0.01 mg_{Pt} cm^{-2} on both sides. The working electrode (hydrogen oxidation reaction) was set to 100% RH and the reference electrode (hydrogen evolution reaction) to 50% and 100% RH for the hydrogen pumping experiments in Figure 7 in the main text. The fully humidified membrane represents the membrane condition while operation of the fuel cell, and the differential humidification created a gradient in the membrane hydration, to pronounce the possible effect of the blocking layer on the water transport in the membrane. Distinct current densities from 0.4 to 1.8 A cm^{-2} were applied and the potential response was detected for 30 min at every current density step. A voltage cut-off at 1 V was set in case of an increasing ohmic resistance of the membrane, which can occur at high current densities due to membrane dehydration.

The increased slope (and therefore resistivity) of the SLG (TT) MEA in comparison to the reference MEA (2 NXL) could be observed slightly at fully humidified conditions and significantly for differential humidification. Furthermore, the voltage cut-off was reached for the SLG (TT) MEA at 1.6 A cm^{-2} under differential humidification, whereas the reference MEA stayed well below this threshold over the whole current density range. Figure 7 and Table S2 show that the resistivity (potential divided by the current density, equal to the slope of the I-V curve) of the reference membrane is significantly lower than the resistivity of the SLG (TT) composite membrane for the differential humidification. For the full humidification case, the trend of a generally higher resistivity and a steeper increase in resistivity with increasing current density for the SLG (TT) membrane is already visible but less pronounced.

Table S2: Current density dependent resistivity of the reference MEA (2 NXL) and the MEA with SLG (TT) composite membrane. The resistivity was calculated from the average potential (last 60 s of a 30 min hold) at distinct current densities from the hydrogen pumping data provided in Figure 7.

Current density / A cm^{-2}	Resistivity (100/100% RH.) 2 NXL / mΩ cm^2	Resistivity (100/100% RH) SLG (TT) / mΩ cm^2	Resistivity (50/100% RH) 2 NXL / mΩ cm^2	Resistivity (50/100% RH) SLG (TT) / mΩ cm^2
0.4	125.0	150.8	209.4	302.2
0.6	125.3	151.7	215.3	314.7
0.8	127.1	152.0	220.7	329.1
1	128.5	156.1	225.6	353.7
1.2	134.0	165.7	234.7	383.3
1.4	141.9	176.2	247.5	431.2
1.6	150.4	187.5	268.4	Potential limit
1.8	160.0	207.0	317.1	

[S1]: Das, A.; Chakraborty, B.; Sood, A. K. Raman spectroscopy of graphene on different substrates and influence of defects. Bull Mater Sci 2008, 31 (3), 579–584. DOI: 10.1007/s12034-008-0090-5.

[S2]: Böhm, T.; Moroni, R.; Breitwieser, M.; Thiele, S.; Vierrath, S. Spatially Resolved Quantification of Ionomer Degradation in Fuel Cells by Confocal Raman Microscopy. *J. Electrochem. Soc.* **2019,** *166* (7), F3044-F3051

[S3]: Specification to product Nafion™ XL, Brand: Chemours; https://5.imimg.com/data5/FQ/TQ/MY-3121249/nafion-membrane-xl.pdf; (accessed December, 2023)
